# Isolation, cloning and expression of *CCA1* gene in transgenic progeny plants of Japonica rice exhibiting altered morphological traits

**DOI:** 10.1371/journal.pone.0220140

**Published:** 2019-08-05

**Authors:** Ashok Chaudhury, Anita Devi Dalal, Nayan Tara Sheoran

**Affiliations:** Plant Molecular Biology Laboratory, Department of Bio and Nano Technology, Bio and Nano Technology Centre, Guru Jambheshwar University of Science and Technology, Hisar, Haryana, India; CSMCRI, INDIA

## Abstract

Circadian clock genes holds tremendous potential for breeding crops better adapted to environmental fluctuations inherent to climate change. Endogenous *TOC1* promoter and *CCA1* gene from rice were isolated, cloned and mobilized into pCAMBIA1300 vectors and RNAi constructs A, B and C. Embryogenic calli of varying ages derived from mature seeds of Taipei 309 were employed for *Agrobacterium*-mediated genetic transformation generating T0, T1 and T2 independent transgenic lines were analyzed for over-expression and repression of *CCA1* gene along with various morphological traits. Six hundred and thirty two T0 transgenic plants were generated from rice calli using constructs A, B and C. T0 progeny plants derived from constructs A, B and C did not show any considerable difference in morphological traits. T1and T2 progeny plants derived from construct A exhibited over-expression of *CCA1* gene, on the contrary, progeny plants derived from RNAi constructs B and C exhibited repression of *CCA1* gene in qRT-PCR analysis at different time points and showed rhythmicity peaking at dawn (6:00 AM) and lowest expression at 12:00 Noon. T1 and T2 progeny plants derived from construct A, namely, A-17 and A-45 exhibited reduced number of tillers/panicles (6–8), reduced thousand seed weight (10.1–16.6g), decreased seed length (4.98 to 6.58mm), decreased seed width (1.1–1.8mm) as compared to wild type plants. T1 and T2 progeny plants of construct B and C showed increased number of tillers/panicles (8–19), better seed yield (4.98–28.9g), increased thousand seed weight (15.6–29.03g), slightly increased seed length (5.7–7.43mm) and slightly increased seed width (1.7–2.98mm) as compared to wild type plants. Chlorophyll content in T1 and T2 progeny plants did not show any significant difference among the three constructs, however, rhythmicity was observed over the period of time in conjunction to *CCA1* gene expression. Evidence has been presented which demonstrates that endogenous repression of *CCA1* gene resulted in improved morphological traits: increased number of tillers/panicle, thousand seed weight, seed size; whereas, over-expression leads to diminution in morphological traits: decreased number of tillers/panicle, thousand seed weight, seed size as compared to the wild type in T1 and T2 progeny plants. This is first report of successful regulation of endogenous *CCA1* gene under control of *TOC1* promoter and its effect on improved growth vigor in Japonica rice.

## Introduction

Circadian Clock Associated1 (*CCA1*) and Late Elongated Hypocotyl (*LHY*) are two important transcription factors that are active and expressed at dawn [1, 2] and they are partially redundant MYB transcription factors that are members of the larger *Reveille* (*RVE*) gene family, which also contains the major clock activators such as RVE8, RVE6, and RVE4 [3–7].

The present day understanding of circadian clock in *Arabidopsis thaliana* is based on the model proposed [8] and later reviewed [9]. It has been proposed that TOC1 increases *LHY/CCA1* expression allowing *LHY/CCA1* expression to reach a peak at dawn beginning the cycle again [8]. In *Arabidopsis* the two MYB transcription factors, *CCA1* and *LHY* [9] together with the PRR 5, 7, 9 jointly function as a Morning Element Loop, both mRNA and post translational level peaking at dawn. It interlocks with an Evening Element Loop comprising of Timing Of Cab Expression 1 (TOC1) or PRR1, GIGANTEA (GI). The Evening Element Loop also contains *Early Flowering* 3 and 4 *(EFF3 & ELF4)* as well as *Lux Arrhythmic (LUX)*. *TOC1* expression oscillates peaking during early evening, opposite to *CCA1* and *LHY*. In particular, the transcription factors *CCA1* and LHY, which are mostly produced in the morning, are thought to repress the expression of the gene that codes for another transcription factor, *TOC1*, which is mostly produced in the evening and, in turn, represses expression of the genes *CCA1* and *LHY*. The cycle starts with light induced *LHY/CCA1* expression at dawn, this results in accumulation of *LHY/CCA1*, which further represses *TOC1* expression which in turn results in reduced activation of *LHY/CCA1*. As the cycle progresses there is a decrease in *LHY/CCA1* expression allowing increase in *TOC1* transcript levels and reach a maximum at dusk, when *LHY/CCA1* are at their lowest. Hayama and Coupland [9] proposed that TOC1 act as activator of *LHY/CCA1* expression by interaction with a basic helix-loop-helix protein, namely, Phytochrome-Interacting Factor3 *(PIF3)*, that in turn binds to *LHY* and *CCA1* promoters. Full length cDNA of *CCA1*, *PRR1* and *ZTL1* genes isolated from Nipponbare rice variety under the control of *CaMv35S* promoter were over expressed in transgenic plants of *Arabidopsis thaliana* by Murakami *et al*. [10]. It was reported that when the rice clock-associated genes were over-expressed in *Arabidopsis thaliana*, it leads to significant perturbation of the endogenous circadian rhythms indicating that circadian clock genes were highly conserved between the two species. *RVE8* activates expression of evening expressed genes such as *TOC1*, *PRR5*, *PRR9*, *GI*, *LUX*, and *ELF4* in opposition to *CCA1* and *LHY* [7, 11]. The *CCA1* has been shown to directly modulate *TOC1* and other downstream genes in circadian clock, photosynthesis and starch metabolism as well as for increased biomass and growth vigor in *Arabidopsis thaliana* [12]. Identification of novel genes against downy mildew disease resistance and its regulation by *CCA1* gene in *Arabidopsis thaliana* was reported [13]. The qRT-PCR of *ZmCCA1* gene was found to express in abundance in maize stem and leaves in morning hours under long day and short day with a rhythmic pattern; whereas, *TOC1* gene was found to express 10-12h after dawn [14]. Over-expression of *ZmCCA1* gene in *Arabidopsis thaliana* resulted in down regulation of GI, Constans (CO) and Flowering locus T (FT) genes leading to longer hypocotyls and delayed flowering.

Later, Huang *et al*. [15] have shown that *TOC1* does not function as an activator but rather functions as a general repressor of oscillator gene expression and repression occurs through *TOC1* rhythmic binding to the circadian oscillator gene promoters. *CCA1* over-expressing (*CCA1-ox*) plants have improved ability to grow in ROS stress-inducing conditions due to the fact that *CCA1* regulates genes involved in production, response, and transcriptional regulation of ROS in *Arabidopsis* has been reported [16]. It has been reported that the *CCA1* gene products are involved in light-regulated transcriptional activation of gene expression. Increased metabolic vigor in developing embryos when the maternal copy of *CCA1* is repressed in inter-specific hybrids of *Arabidopsis* was reported [17]. Wheels within wheels: the plant circadian system has been reviewed and documented [18]. It has been reported that the *CCA1* gene products are involved in light-regulated transcriptional activation of gene expression. *CCA1* genes universally control a number of vital plant activities, including development, growth, and reproduction and key agricultural traits [19]. Rice has become a model system for studying gene expression and regulation [20]. So far there has been no report on studies on regulation of endogenous *CCA1* gene in transgenic plants of rice. Keeping this in view, gene constructs *pCTCN* for over-expression vector (A) and RNAi vectors *pCTSaASN* and *pCTSbASN* for repression (B and C) have been generated using standard molecular biology protocols in the plasmid vector pCAMBIA 1300 (8.959kb). Transgenic plants of Japonica rice variety Taipei 309 plants using *Agrobacterium tumefaciens*, strain EHA 105 harboring the above three constructs have been generated by introducing endogenous *CCA1* gene under the control of *TOC1* promoter for studying *CCA1* gene expression in both up-regulated and down-regulated manner in T0, T1 and T2 progeny plants and the morphological characteristics were analyzed.

## Materials and methods

### Construction of plasmid vectors pCTCN, pCTSaASN and pCTSbASN

#### Isolation, cloning of *TOC1* promoter, *NOS* terminator and *CCA1* gene in *PUC19*

Seeds of rice variety Taipei 309 were germinated in pots in a BOD incubator at 25°C for two weeks. Genomic DNA was isolated using Qiagen DNAEasy plant mini kit by as per manufacturer’s protocol by quickly freezing 500–800 mg tissue (shoot) in liquid nitrogen and ground to a fine powder in a pestle & mortar. Washed the pellet with 500 μl 95% ethanol at 8,000 rpm for 1 min; discard the flow through and spin again for 2 min to get rid of traces of ethanol. Transfer the DNAEasy spin column into a new 1.5 ml eppendorf tube, let it dry for 1.0 min at room temperature, added 200 μl of sterile distilled water and centrifuge at 13,000 rpm for 5 min to recover the genomic DNA. Genomic DNA was quantified by Nano Drop Spectrophotometer.

The 1.35 kb *TOC1* gene promoter has been PCR amplified using primers Forward 5’-TAT AAA GCT TAC TCC AAG CTC CTG CTA CTG-3’ and Reverse 5’-ATA TGG ATC CCC TAC CTT TTG CTT TCC TCT-3’ using AccuPower PCR premix kit (Bioneer) in a final volume of 25 μl from genomic DNA of rice variety Taipei 309 germinated seeds. The 5’ forward (*HindIII*) and 5’ reverse (*BamHI*) primers were designed for Rice *TOC1* (Os02g0618200) gene promoter region (02g 25426077–25427419) from NCBI. The PCR cycle comprised of Hot start 94°C 3 min, Denaturation 94°C 30 sec, Annealing 55°C 30 sec, Extension 72°C 1 min thirty five cycles, followed by 72°C 10 min and 4°C hold using AccuPower PCR premix kit (Bioneer) in a final volume of 25 μl. The amplicon was gel extracted using Qiaquick kit and cloned into multiple cloning site of pUC19 vector using *HindIII* and *BamHI* restriction enzymes.

The *NOS* terminator gene was PCR amplified using primers NOS Forward 5’TAA GTG AGC TCG ATC ACG CGT TCT AG-3’ and NOS Reverse 5’-ACT GCG AAT TCC GTA CAT GGT CGA TA-3’ using AccuPower PCR premix kit (Bioneer) in a final volume of 25 μl. The amplicon was gel extracted using Qiaquick kit and digested with *SacI* and *EcoRI* cloned into *pUC19* having *TOC1* gene to yield pUCTOC1NOS. The 5’ forward (*SacI*) and 5’ reverse (*EcoRI*) primers were designed for *NOS* terminator gene. The PCR cycle comprised of Hot start 94°C 3 min, Denaturation 94°C 30 sec, Annealing 55°C 30 sec, Extension 72°C 1 min thirty five cycles, followed by 72°C 10 min and 4°C hold using AccuPower PCR premix kit (Bioneer) in a final volume of 25 μl.

Although the *CCA1* gene sequence has been amplified from rice genome as template, the restriction enzyme recognition sites (employed for cloning) were killed/knocked out without altering the original amino acid sequence CCA1 protein. The 2.172 kb *CCA1* gene from rice Os08g0157600 (original) from NCBI was modified in which codon usage has been optimized for plants; and certain restriction enzyme sites have been killed as shown in S1 Fig. Modified *CCA1* gene was got synthesized from GenScript USA Inc., Piscataway, NJ, USA as a cDNA clone in pUC57 vector; the *CCA1* gene was excised out using *SacI* and *BamHI* and it was cloned into *pUC19* having *TOC1* promoter and *NOS* gene described above giving *PTCN* vector (PUC19:TOC1:CCA1:NOS).

#### Isolation, cloning of *CCA1a* and *CCA1b* gene for construction of RNAi vectors

The *CCA1a* and *CCA1b* sense and antisense fragments for making the two RNAi constructs pCTSaASN and pCTSbASN were designed by choosing 400 bp of N terminal region of *CCA1*a gene and 395 bp of C terminal region of *CCA1b* gene, respectively as shown in S1 Fig. PCR amplification for *CCA1a* sense *BamHI-XHoI* (400bp) using gene specific primers Forward 5’-GCT AGG ATC CTC CTC TGG TGA GGA A-3’ and Reverse 5’-TAG CCT CGA GCC ATT TGT GCA GTG C-3’ and *CCA1a* antisense *SacI-XmaI* (400bp) using gene specific primers Forward 5’-GCT AGA GCT CTC CTC TGG TGA GGA A-3’ and Reverse 5’-TAG CCC CGG GCC ATT TGT GCA GTG C-3’. PCR amplification for *CCA1b* sense *BamHI-XHoI* (395bp) using gene specific primers Forward 5’- GCT AGG ATC CGA GAA AGA TAT AGA C -3’ and Reverse 5’- TAG CCT CGA GGT GCT TGC ACT GCT C-3’ and *CCA1b* antisense *SacI-XmaI* (395bp) using gene specific primers Forward 5’- GCT AGA GCT CGA GAA AGA TAT AGA C-3’ and Reverse 5’- TAG CCC CGG GGT GCT TGC ACT GCT C-3’. The amplifications conditions were same as described for *TOC1* promoter using AccuPower PCR premix kit (Bioneer) in a final volume of 25 μl.

The amplified products of *CCA1a* sense and *CCA1a* antisense genes were digested with *BamHI*, *XhoI* and *XmaI*, *SacI*, respectively, and cloned into the intermediate RNAi vector psd20 by kicking out the *4CL* gene in sense and antisense orientation linked with a GUS linker. Similarly, the amplified products of *CCA1b* sense and *CCA1b* antisense genes were digested with *BamHI*, *XhoI* and *XmaI*, *SacI*, respectively, and cloned into the intermediate RNAi vector psd20 by kicking out the *4CL* gene in sense and antisense orientation linked with a GUS linker. The cloning of *CCA1a* sense and *CCA1a* antisense genes in intermediate RNAi vector psd20 involved two steps of sequential cloning by first ligating *CCA1a* sense gene by digesting with *BamHI*, *XhoI*, followed by cloning of *CCA1a* antisense gene using *XmaI*, *SacI* restriction enzymes in which *CCA1a* sense fragment has already been moved in. Similarly, the cloning of *CCA1b* sense and *CCA1b* antisense genes in intermediate RNAi vector psd20 involved two steps of sequential cloning by first ligating *CCA1a* sense gene by digesting with *BamHI*, *XhoI*, followed by cloning of *CCA1b* antisense gene using *XmaI*, *SacI* restriction enzymes in which *CCA1b* sense fragment has already been moved in.

The *CCA1a* sense:*GUS* linker:*CCA1a* antisense fragments was excised out using *BamHI SacI* and cloned into pUCTOC1NOS vector by ligating into *BamHI SacI* site to yield *pUCTSaASN* vector. Similarly, the *CCA1b* sense:*GUS* linker:*CCA1b* antisense fragments were excised out using *BamHI SacI* and cloned into pUCTOC1NOS vector by ligating into *BamHI SacI* site to yield pUCTSbASN vector.

#### Isolation, cloning of over-expression vector pCTCN and RNAi vectors pCTSaASN and pCTSbASN for repression in pCAMBIA1300

The *HindIII EcoRI* fragment having *TOC*+*CCA1*+*NOS* gene from this *pUC19* intermediate vector *PTCN* was digested using *HindIII* and *EcoRI*, purified using Qiaquick kit and cloned into pCambia1300 vector to give over-expression construct *pCTCN (A)* using standard molecular biology protocols.

The *HindIII EcoRI* fragment from the *pUC19* intermediate vector (pUCTSaASN) having *TOC+CCA1a* sense *GUS linkerCCA1a* antisense *+NOS* was purified and cloned into pCambia1300 vector to give *pCTSaASN* (B) construct. Similarly, the *HindIII EcoRI* fragment from this pUC19 intermediate vector *(pUCTSbASN)* having *TOC+CCA1b* sense *GUS linkerCCA1b* antisense *+NOS* was purified and cloned into pCambia1300 vector to give pCTSbASN (C) construct.

These three constructs A, B and C were then mobilized into chemically competent *Agrobacterium tumefaciens* strains EHA105 cells. Competent cells were prepared by streaking on LB + Rif 25 mgl^-1^ agar plates and incubated at 28°C for obtaining single colonies. Single colony was picked and grown in 5 ml YEP medium + Rif at 28°C, 200 rpm for 2 days. Five ml of this active culture was added to 50 ml YEP medium + Rif and grown as above (O/N) till the OD600 reaches 0.7. Chill the culture on ice. Centrifuged the cell suspension at 5000 rpm at 4°C for 5 min in 50ml yellow cap tubes. Discard the supernatant; resuspend the cells in 1.0 ml of ice cold 20mM CaCl_2_. Dispense 0.1ml aliquots into pre-chilled eppendorf tubes. The cells should be frozen in liquid in nitrogen and stored at -80°C for further use. For transformation added 1 μg of plasmid DNA A, B and C constructs and freeze the cells in liquid nitrogen. Thaw the cells by incubating the tubes at 37°C for 5 min. Add 300μl of YEP and incubate at 28°C with gentle shaking at 100–120 rpm to allow the antibiotic resistance gene to express. Spread on YEP + Rif (20 mgl^-1^) + Kan (50 mgl^-1^) agar plates incubate at 28°C by inverting them for three days for appearance of single colonies.

Single colonies from all the three constructs A, B and C mobilized in *Agrobacterium tumefaciens* were grown in 5 ml YEP cultures and plasmid DNA was isolated using Qiagen mini prep kit. The DNA was eluted in 100 μl of sterile water and quantified using Nano Drop Spectrophotometer. The presence of cloned gene constructs was confirmed by performing PCR amplification using 2μl of the isolated plasmid DNA of all the three constructs using primers for *TOC1* gene promoter.

### Callus induction

Dehusked seeds of rice variety Taipei 309 were surface sterilized with 1:1 Clorox bleach (6.0% sodium hypochlorite) and water in 100 ml total volume for 20 min, washed several times with sterile distilled water. Fifteen to twenty seeds were inoculated on N6 callus induction medium supplemented with 250 mgl^-1^ Myo-inositol, 1.0gl^-1^ Casein hydrolysate, 690 mgl^-1^ Proline, 1.0mgl^-1^ Thiamine HCl, 30gl^-1^ Maltose, 3gl^-1^ Phytagel, pH 5.8 [21, 22] autoclaved and upon cooling added 5.0 mgl^-1^ 2,4-D and 0.1 mgl^-1^ BAP and dispense in 90 mm Petri dishes and incubated in dark at 25±2°C. The calli was separated from germinating seed after removing the shoot and root axis and sub-cultured on to fresh callus induction medium every two weeks. Detailed composition of various media used for tissue culture, *Agrobacterium*-mediated genetic transformation studies are given in Table 1.

**Table 1:** Composition of various media used for tissue culture and *Agrobacterium*-mediated genetic transformation studies.

Culture medium	Culture Time	Constituents
**Callus induction medium**	3–4 weeks	3.98gl^-1^ Chu N6 Salt Caisson Cat CHP01; 0.250gl^-1^ Myo inositol; 690mg l^-1^ Proline; 1 g l^-1^ Casein Hydrolysate (CH); 30gl^-1^ Maltose, 5.0mgl^-1^ 2,4-D; 0.1mgl^-1^ BAP; 3g l^-1^ Phytagel, pH 5.8
**Infection Medium**	15 min	4.33 g MS Salt Caisson Cat MSP01; 0.250 g Myo inositol; 690 mg l^-1^ Proline; 1.0 g l^-1^ Casein Hydrolysate (CH); 30gl^-1^ Glucose; 5.0 mgl^-1^ 2,4-D; 0.1 mg l^-1^ BAP; 200 μM Acetosyringone; 3gl^-1^ Phytagel, pH 5.2
**Co-cultivation medium**	2 days	3.98gl^-1^ Chu N6; 0.250gl^-1^ Myo-inositol; 690 mgl^-1^ Proline; 1 gl^-1^ Casein Hydrolysate (CH); 30gl^-1^ Maltose, 0.5mgl^-1^ 2,4-D; 0.1mgl^-1^ BAP; 200 μM Acetosyringone; 3gl^-1^Phytagel, pH 5.8
**Selection medium**	6 weeks	3.98gl^-1^ Chu N6 Salt; 0.250gl^-1^ Myo inositol; 690 mgl^-1^ Proline; 1.0gl^-1^ Casein Hydrolysate (CH); 30 g l^-1^ Maltose, 5.0mgl^-1^ 2,4-D; 0.1 mg l^-1^ BAP; 3.0gl^-1^ Phytagel, 200mg l^-1^ Timentin; 50mgl^-1^ Hygromycin; pH 5.8
**Regeneration medium (RM-I)**	4–6 weeks	4.33gl^-1^ M S salts; 30gl^-1^ Maltose; 3gl^-1^ Phytagel; 2mgl^-1^ BAP; 200 mgl^-1^ Timentin; 50 mgl^-1^ Hygromycin; pH 5.8
**Rooting medium (RM-II)**	2 weeks	2.15gl^-1^ M S salts 30gl^-1^ Maltose; 3g l^-1^ Phytagel; 200 mgl^-1^ Timentin; 50mgl^-1^ Hygromycin; pH 5.8

### *Agrobacterium*-mediated genetic transformation

Embryogenic calli obtained from mature seeds of Taipei 309 were employed for *Agrobacterium*-mediated genetic transformation of rice calli of varying ages from one month to five months as per protocol reported by Patel *et al*. [22] with minor modifications. The four constructs, namely, A, B & C mobilized in *Agrobacterium tumefaciens*, and pCUbiGFP glycerol stocks was used to inoculate in 5 ml of YEP medium supplemented with Rif (20 mgl^-1^) + Kan (50 mgl^-1^) incubate at 28°C with continuous shaking at 200 rpm overnight. Next day morning transfer all the 5 ml culture in 45 ml Infection media having MS basal media salts supplemented with 1 mgl^-1^ Thiamine HCl, 250 mgl^-1^ Myoinositol, 1.0 gl^-1^ Casein hydrolysate, 690 mgl^-1^ Proline, 30 gl^-1^ Glucose, 5.0 mgl^-1^ 2,4-D and 200 μM Acetosyringone, pH 5.2 and grown at 28°C with continuous shaking at 200 rpm for 2–4 h till the OD_600_ reaches 0.5–0.6. Infected the embryogenic calli with the *Agrobacterium* cells by giving heat shock at 42°C for 3 min followed by incubation of 12 min at room temperature, excess of suspension drained off and the calli were blotted on to five layers sterile Whatman papers to remove excessive *Agrobacterium* suspension and co-cultivated for two days on co-cultivation media having 200 μM Acetosyringone at 25°C in dark.

### Selection and regeneration of T0 transformed plants

On third day the calli were transferred on to Selection media (same as callus induction media) supplemented with 200mgl^-1^ Timentin and 50mgl^-1^ hygromycin and incubated at 25°C in dark for two weeks. Three more transfers were made on to fresh selection media after every two weeks by transferring newly induced calli, and discarded the brown or *Agrobacterium* infected calli. The transformed calli was very slow growing creamy white to yellow in color and very light weight. After eight weeks of selection the calli growing on hygromycin was transferred to Regeneration media, MS media supplemented with 2.0 mgl^-1^ BAP, 30 gl^-1^ Maltose, 3.0 gl^-1^ Phytagel, 200 mgl^-1^ Timentin and 50 mgl^-1^ hygromycin and incubated at 25°C in light for two to three weeks. The calli was transferred to fresh Regeneration media (RM-I) for another two to three weeks for inducing shoots. The regenerated plants were transferred to MS basal media (RM-II) with 200mgl^-1^ Timentin and 50mgl^-1^ hygromycin for further proliferation and rooting in Magenta boxes at 25°C in light. The primary transgenic plants 8-10cm with well developed shoot and root growing on MS basal media supplemented with 200 mgl^-1^ Timentin and 50 mgl^-1^ hygromycin in Magenta boxes were carefully removed, washed in running tap water to remove adhering agar were planted in 6 inch pots in 50:50 peat-lite/sand in green house maintained at 26/22°C with 12h day/night regime and high humidity. Six pots were placed in individual plastic trays half filled with standing water. The plants were initially covered with a glass beaker and the cart was covered with a shade net for acclimatization and for preventing transpiration loss. After 3–4 weeks the transgenic plants were shifted to plant growth chambers maintained at 28/24°C with 12h day/night regime for harvesting T0 seed in Phytotron. The plants were watered and given nutrients as per standard management practice.

### DNA isolation and PCR analysis of T0, T1 and T2 transgenic progeny plants

The genomic DNA was isolated from young leaf tissues of randomly selected five primary transgenic T0 plants as well as T1 and T2 transgenic progeny plants for over-expression of *CCA1* gene constructs A, and repression constructs B & C and wild type (WT) by using modified CTAB method according to [23]. The plasmid DNA was isolated from *Agrobacterium* strains using a rapid mini-prep method according to [24]. The plasmid DNA amplified with *hyg II* gene specific primers was used as positive control. PCR was carried out as the first method to confirm the transgenic nature of the regenerated plants and T1 and T2 progeny plants as described [25]. PCR analysis was performed using 100ng of genomic DNA (for plasmid DNA 5ng) in a 25μl reaction mixture with two sets of *hyg II* gene specific primers, one set *HygF1* 5’-CGA AAT TGC CGT CAA CCA AGC TCT-3’, *HygR1* 5’-AGG CTC TCG ATG AGC TGA TGC TTT-3’ and second set of primers *HygF2* 5’-CGC GCA TAT GAA ATC ACG CCA TGT-3’), *HygR2* 5’-ATA GCT GCG CCG ATG GTT TCT ACA-3’. The hygromycin sequence in total DNA was amplified using AccuPower PCR premix kit (Bioneer). The PCR cycle comprised of pre-incubation period at 94°C for 3 min, leading to 35 cycles of denaturation at 94°C for 1 min, annealing at 60°C for 1 min and synthesis at 72°C for 1 min, followed by extension at 72°C for 5 min. The amplified PCR product (10μl) was subjected to electrophoresis on a 1% agarose gel and visualized under UV Transilluminator.

### Southern blot analysis of T0 transgenic progeny plants

For the verification of the stable integration of *hyg* gene, genomic DNA isolation was performed from leaves of T0 rice plants, digested with *Hind III* restriction enzyme, subjected to agarose gel electrophoresis and Southern blot analysis was done using DIG High prime DNA Labeling and Detection starter Kit I, Sigma as per protocol reported by^39^ with minor modifications. For Southern blot analysis, 10 μg of DNA was digested with *Hind III* and subjected to electrophoresis on 1.0% agarose and blotted on to a nylon membrane (Immobilion N^+^, Millipore Corporation, MA, U.S.A.) by capillary blotting. The membrane was UV cross-linked and probed with DIG labeled 700bp *hyg* gene coding region. Hybridization was carried out at 42°C. All other procedures were performed according to the manufacturer’s instructions.

### Raising of T1 and T2 transgenic progeny plants from T0 and T1 seeds

The T0 transgenic rice seeds were harvested after maturity in Phytotron, N C State University, Raleigh, North Carolina, USA. The T0 seeds were imported with the permission of the Department of Biotechnology, Ministry of Science of Technology, Government of India, New Delhi, India through National Bureau of Plant Genetic Resources, New Delhi, India. The T0 seeds derived from three constructs A, B and C and wild type plants (WT) were raised in 6 inches pots in 50:50 peat-lite/sand in Transgenic Green House maintained at 26/22°C with 12h day/night regime and high humidity as described earlier to obtain T1 transgenic progeny plants with standard agronomic and management practices in a completely randomized block design with five independent lines of each of the constructs in the Transgenic Green House, Department of Bio & Nano Technology, Guru Jambheshwar University of Science and Technology, Hisar, Haryana, India and T1 seeds were harvested. The T0 lines which showed single copy insertion of *Hyg* gene in Southern analysis were chosen to raise T1 progeny plants. The plants which were performing morphologically better, were selected for raising T1 progeny plants and similar trend was followed in case of T2 progeny plants from T1 seeds. Similarly, T1 seeds derived from three constructs A, B and C and WT plants were raised in 6 inches pots in 50:50 peat-lite/sand in Transgenic Green House maintained at 26/22°C with 12h day/night regime and high humidity to obtain T2 transgenic progeny plants in a completely randomized block design with five independent lines of each of the three constructs A, B and C in the Transgenic Green House and T2 seeds were harvested.

### Collection of morphological data of T0, T1 and T2 transgenic progeny plants and statistical analysis

Morphological data for transgenic progeny plants for over-expression construct A, and repression constructs B & C and wild type (WT) for plant height, numbers of tillers/panicles, yield, thousand seed weight, seed length, seed width and chlorophyll content was collected. The data was subjected to statistical analysis using Student’s *t*-test. All the morphological data of T1 and T2 transgenic progeny is presented as the mean ± SE and *P* value to compare the obtained parameters from transgenic lines (TG) and wild type plant (WT) and a *P* value of < 0.05, was considered to be statistically significant.

### *CCA1* Gene expression analysis of T0, T1 and T2 transgenic progeny plants

Transgenic plants harboring gene constructs for *CCA1* gene in over-expression and repression mode were grown in Phytotron/Transgenic Green House for 4 weeks in 12/12-h (day/night) cycles for 24 h *CCA1* rhythms analysis and transgenic plants/lines which were found to be PCR positive for *hyg* gene were chosen from T0, T1 and T2 Transgenic progeny plants. For each genotype, mature leaves from five transgenic plants for each constructs namely, A, B and C and wild type (WT) were collected at 6:00AM, 12:00 Noon, 6:00 PM and 9:00 PM and frozen in liquid nitrogen. Analysis for *CCA*1gene expression was performed on samples collected at 6:00 AM, 12:00 Noon, 6:00 PM and 9:00 PM using Real time PCR (Applied-Biosystem).

### Extraction of total RNA and quantitative real time-PCR analysis of T0, T1 and T2 transgenic progeny plants

Total RNA was extracted from young leaves of various lines of transgenic rice plants derived from *Agrobacterium*-mediated genetically transformed constructs A, B and C and wild type plants using Trizole based method of RNA isolation. First-strand cDNA synthesis was done using Affinity Script Multiple Temperature First—strand cDNA synthesis Kit, and Promega (Go Script Reverse Transcription System Agilent Genomics). Transcript levels of each gene were measured by real-time quantitative qRT-PCR was carried out with Step One Real-time PCR system (Applied Biosystems). Quantitative PCR mixture for expression of plant *CCA1* gene was prepared in MicroAmp Fast Optical 96 well reaction plate according to the manufacturer’s protocols. The gene-specific primers used for qRT-PCR for *CCA1A* gene were Forward 5’-TTT CGA GAA GTC CCA TCG GCA TCA-3’ and Reverse 5’-TTT GCA TCC TTC CCT GCA CCA TTG-3’ and Forward 5’-GGC TCA AGC CGA TGG AAG-3’ and Reverse 5’-AGC ACG ACA GGG TTT AAC AAG-3’ for 18 *S rRNA* as internal control (GenBank Accession No. AF156675). The level of the *CCA1* gene expression in T0, T1 and T2 transgenic plants was determined by real-time PCR following the 2^-ΔΔCT^ method of Livak and Schmittgen [26] at different time points. The qRT-PCR experiment was performed in triplicate.

### Quantification of chlorophyll content in T1 and T2 transgenic progeny plants

Quantification of Chlorophyll from various lines of T1and T2 transgenic progeny plants derived from three constructs A, B and C as per protocol of Hiscox and Israelstam [27] with slight modifications. Briefly, 100 mg of transgenic plant leaf tissue were taken at various time points 6:00AM, 12:00 Noon, 6:00 PM and 9:00 AM the following day, and placed in 12 ml vial containing 7 ml DMSO (Dimethyl Sulphoxide) and incubated at 65^*°*^C for 30 min. After incubation, samples were transferred to a 25 ml graduated tube and the volume was made to 10 ml with DMSO 1 ml of above sample was transferred to a glass cuvettes. Spectrophotometer was calibrated against DMSO at 645nm and 663nm (Chlorophyll method). Absorbance was recorded at 645 and 663 nm and chlorophyll content was calculated using equation suggested by Arnon [28] given below:-

Chlorophyll a (mg g^-1^) = 0.0127 × A663–0.00269 × A645

Chlorophyll b (mg g^-1^) = 0.0229 × A645–0.00468 × A663

Total Chlorophyll a+b (mg g^-1^) = 0.0202× A645–0.00802 × A663

## Results & discussion

### Isolation, cloning of *TOC1* promoter, *NOS* terminator and *CCA1* gene in PUC 19

In the present investigation endogenous *CCA1* gene under the control of *TOC1* gene promoter have been isolated, cloned from *Japonica* rice and mobilized into pCAMBIA1300 vectors and RNAi constructs A, B and C have been employed for *Agrobacterium*-mediated genetic transformation of embryogenic call derived from rice variety Taipei 309 have been analyzed for altered morphological traits as well as *CCA1* gene expression in T0, T1 and T2 transgenic progeny plants. These two essential genes, namely *TOC1* and *CCA1* genes have been chosen in the present investigation because; *TOC1* gene is the master controller of various circadian clock genes; simultaneously, *CCA1* gene products are known for controlling a large number of important plant’s attributes: growth, development, flowering, reproduction and many other important agricultural traits. The TOC1 is a Transcription Factor which is under circadian control and involved in regulation of its own feed-back-loop and part of the five Pseudo-Response-Regulators (PRR) in plants. TOC1 is known to contribute to plant fitness in carbon fixation and biomass production. That’s why TOC1 promoter was chosen over *CCA1* gene own promoter. The 1.35 kb *TOC1* gene promoter has been PCR amplified using primers using genomic DNA obtained from two weeks old germinated seeds of rice variety Taipei 309 as shown in S2A Fig. The 5’ forward (*HindIII*) and 5’ reverse (*BamHI*) primers were designed for Rice *TOC1* (Os02g0618200) gene promoter region (02g 25426077–25427419) from NCBI. The amplicon was gel extracted using Qiaquick kit and cloned into multiple cloning site of pUC19 vector using *HindIII* and *BamHI* restriction enzymes. Six transformed colonies were selected, plasmid mini prep was performed, digested with *HindIII* and *BamHI* and the 1–6 clones were found to have 1.35kb *TOC1* gene promoter as shown in S2B Fig. The *NOS* terminator gene was PCR amplified using primers described in Methods and shown in S2C Fig. The 250 bp *NOS* gene amplicon was gel extracted using Qiaquick kit and digested with *SacI* and *EcoRI* cloned into pUC19 having *TOC1* gene to yield pUCTOC1NOS. The 5’ forward (*SacI*) and 5’ reverse (*EcoRI*) primers were designed for *NOS* terminator gene. The 2.172 kb Modified *CCA1* gene which was got synthesized from GenScript USA Inc., Piscataway, NJ, USA as a cDNA clone in pUC57 vector; the *CCA1* gene was excised out using *SacI* and *BamHI* and it was cloned into pUC19 having *TOC1* promoter and *NOS* gene described above giving PTCN vector (PUC19:TOC1:CCA1:NOS). The three clones (1–3) had the 2.172 kb *CCA1* gene as shown in S2D Fig. Similarly, [14] reported cloning and expression of *CCA1* gene in maize (*ZmCCA1*) and revealed that the *ZmCCA1* transcript was highly homologous with *AtCCA1* from *Arabidopsis* and *OsCCA1* from rice [12].

### Isolation, cloning of *CCA1a* and *CCA1b* gene for construction of RNAi vectors

The RNAi is a homology dependent gene silencing technology through RNA cleavage or DNA methylation. The sense and antisense fragment against the target gene, say, *CCA1* forms a hairpin stem loop dsRNA structure which acts as a substrate for the Dicer. Since the entire fragment of *CCA1* gene (2.172kb) of rice as shown in S1 Fig. is quite large and has not been used for construction of RNAi vectors in sense and antisense orientation for complete inhibition, but instead 5`and 3`ends of *CCA1* gene have been chosen in order to assess any variation in efficiency of gene silencing. Therefore, in the present investigation sense and antisense fragments have been selected as; one 400bp fragment designated as *CCA1a* derived from 5`region of *CCA1* gene and second 395bp fragment designated as *CCA1b* derived from 3`region of *CCA1* gene were amplified as described below. It is known that the length and the sequence of the stem region comprising the sense and antisense fragments directly control the efficiency of gene silencing in RNAi technology. Wesley *et al*. [29] reported that RNAi constructs having sense and antisense fragments of complete cDNA of *FLC1* gene (600bp) or the two third part of 3`region (400bp) of *FLC1* gene showed early flowering in transgenic *Arabidopsis*. On the other hand, Heilersig *et al*. [30] reported that RNAi constructs having sense and antisense fragments from 5`region (488bp) of *GBSS1* rice starch synthase gene is more efficient in gene silencing than the middle or 3`region of the target gene in transgenic potato. Therefore, two fragments were derived from PCR amplification for *CCA1a sense BamHI-XHoI* (400bp) lane 2, *CCA1a* antisense *SacI-XmaI* (400bp) lane 3; and *CCA1b sense BamHI-XHoI* (395bp) lane 4 and *CCA1b* antisense *SacI-XmaI* (395bp) lane 5 and 6 representing the 5`and 3`region of *CCA1* gene using gene specific primers was performed as shown in S3A Fig.

The amplified products of *CCA1a* sense and *CCA1a* antisense genes were digested with *BamHI*, *XhoI* and *XmaI*, *SacI*, respectively, and cloned into the intermediate RNAi vector psd20 by kicking out the *4CL* gene in sense and antisense orientation linked with a GUS linker as shown in S3B Fig. Similarly, the amplified products of *CCA1b* sense and *CCA1b* antisense genes were digested with *BamHI*, *XhoI* and *XmaI*, *SacI*, respectively, and cloned into the intermediate RNAi vector psd20 by kicking out the 4CL gene in sense and antisense orientation linked with a GUS linker.

The cloning of *CCA1a* sense and *CCA1a* antisense genes in intermediate RNAi vector psd20 involved two steps of sequential cloning by first ligating *CCA1a* sense gene by digesting with *BamHI*, *XhoI*, followed by cloning of *CCA1a* antisense gene using *XmaI*, *SacI* restriction enzymes in which *CCA1a* sense fragment has already been moved in as shown in S3C and S3D Fig. Out of six clones, clones 1, 2 and 5 gave the correct size (400bp) of *CCA1a* sense gene. Whereas, out of 9 clones 1, 2, 3, 5, 6, 8 showed the correct size (400bp) of *CCA1a* antisense gene. Similarly, the cloning of *CCA1b* sense and *CCA1b* antisense genes in intermediate RNAi vector psd20 involved two steps of sequential cloning by first ligating *CCA1a* sense gene by digesting with *BamHI*, *XhoI*, followed by cloning of *CCA1b* antisense gene using *XmaI*, *SacI* restriction enzymes in which *CCA1b* sense fragment has already been moved in as shown in S3E and S3F Fig. Out of eight clones, all the clones except clone 5 gave the correct size (395bp) of *CCA1b* sense gene. Whereas, on the other hand, out of eight clones, all the clones except clone 7 gave the correct size (395bp) of *CCA1b* antisense gene.

**Fig 1:**
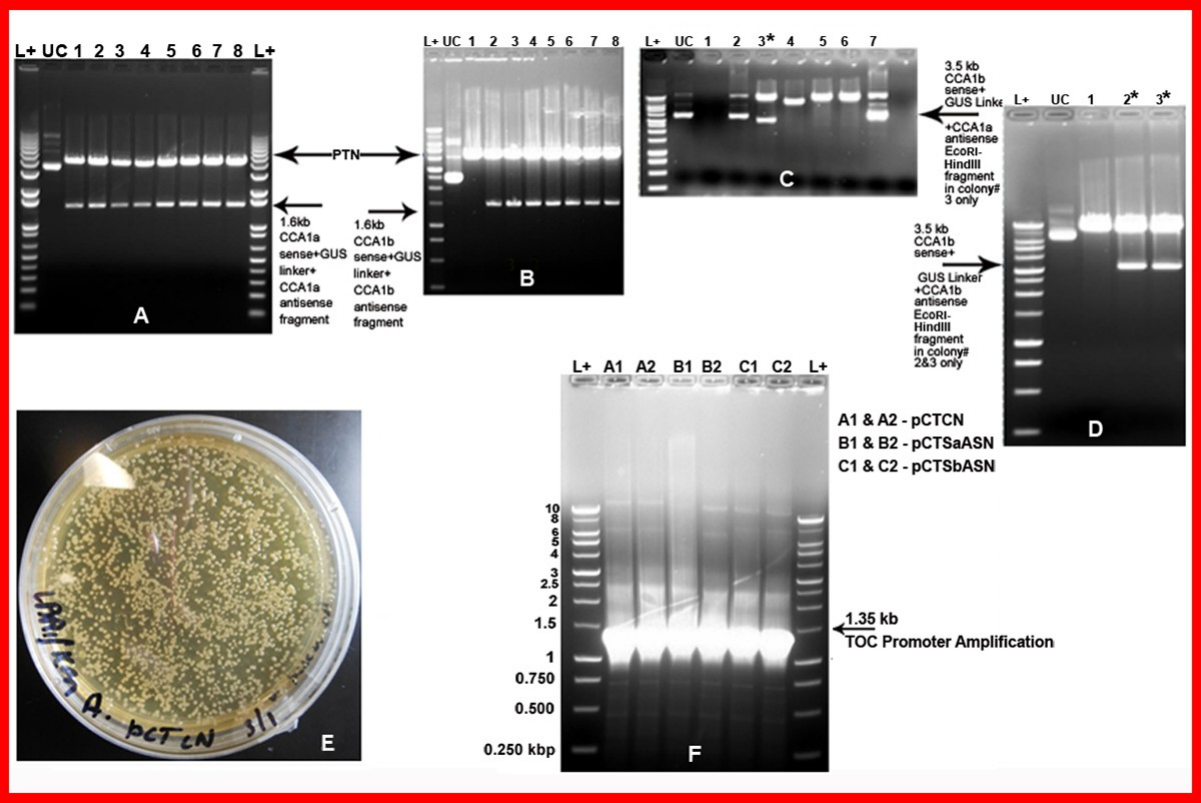
Isolation, cloning of over-expression vector pCTCN and RNAi vectors pCTSaASN and pCTSbASN for repression in pCAMBIA1300. (A) Cloning of *CCA1a* sense:GUS linker:*CCA1a* antisense fragments was excised out using *BamHI SacI* and cloned into pUCTOC1NOS vector by ligating into *BamHI SacI* site to yield pUCTSaASN vector. (B) *CCA1b* sense:GUS linker:*CCA1b* antisense fragments were excised out using *BamHI SacI* and cloned into pUCTOC1NOS vector by ligating into *BamHI SacI* site to yield pUCTSbASN vector. (C) The *HindIII EcoRI* fragment from the pUC19 intermediate vector (pUCTSaASN) having *TOC+CCA1a sense GUS linkerCCA1a antisense +NOS* was purified and cloned into pCambia1300 vector to give pCTSaASN named as construct B. (D) The *HindIII EcoRI* fragment from this pUC19 intermediate vector (pUCTSbASN) having *TOC+CCA1b sense GUS linkerCCA1b antisense +NOS* was purified and cloned into pCambia1300 vector to give pCTSbASN named as construct C. (E) The three constructs A, B and C were then mobilized into chemically competent *Agrobacterium tumefaciens* strains EHA105 cells and single colonies were obtained on LB + 25 mgl^-1^ Rif + 50 mgl^-1^ Kan designated as pCTCN for Construct A. (F) PCR amplification and confirmation of 1.35 kb *TOC1* band from randomly selected colonies A1, A2, B1, B2, C1 and C2 RNAi constructs from each of the three plates having constructs A, B and C mobilized in *Agrobacterium tumefaciens*.

The *CCA1a* sense:GUS linker:*CCA1a* antisense fragments was excised out using *BamHI SacI* and cloned into pUCTOC1NOS vector by ligating into *BamHI SacI* site to yield pUCTSaASN vector. All the eight clones (1–8) gave 1.6 kb fragment having *CCA1a* sense fragment + GUS linker + *CCA1a* antisense fragment along with remaining pUCTOC1NOS fragment as shown in Fig 1A. Similarly, the *CCA1b* sense:GUS linker:*CCA1b* antisense fragments were excised out using *BamHI SacI* and cloned into pUCTOC1NOS vector by ligating into *BamHI SacI* site to yield pUCTSbASN vector. All the eight clones (1–8) except clone1, gave 1.6 kb fragment having *CCA1b* sense fragment + GUS linker + *CCA1b* antisense fragment along with remaining pUCTOC1NOS fragment as shown in Fig 1B.

### Isolation, cloning of over-expression vector pCTCN and RNAi vectors pCTSaASN and pCTSbASN for repression in pCAMBIA1300

The *HindIII EcoRI* fragment having *TOC*+*CCA1*+*NOS* gene from this pUC19 intermediate vector PTCN was digested using *HindIII* and *EcoRI*, purified using Qiaquick kit and cloned into pCambia1300 vector to give over expression construct pCTCN (A) using standard molecular biology protocols.

The *HindIII EcoRI* fragment from the pUC19 intermediate vector (pUCTSaASN) having *TOC+CCA1a sense GUS linkerCCA1a antisense +NOS* was purified and cloned into pCambia1300 vector to give pCTSaASN (B) construct. Out of seven clones, only clone 3 gave 3.5 kb *CCA1a* sense fragment + GUS linker + *CCA1a* antisense fragment after *EcoRI HindIII* digestion as shown in Fig 1C. Similarly, the *HindIII EcoRI* fragment from this pUC19 intermediate vector (pUCTSbASN) having *TOC+CCA1b sense GUS linkerCCA1b antisense +NOS* was purified and cloned into pCambia1300 vector to give pCTSbASN (C) construct. Out of three clones, clone 2 and 3 gave 3.5 kb *CCA1b* sense fragment + GUS linker + *CCA1b* antisense fragment after *EcoRI HindIII* digestion as shown in Fig 1D. These three constructs A, B and C were then mobilized into chemically competent *Agrobacterium tumefaciens* strains EHA105 cells and single colonies were obtained on LB + 25 mgl^-1^ Rif + 50 mgl^-1^ Kan as shown in Fig 1E for pCTCN (A), similar results were obtained for the other two RNAi constructs, namely, pCTSaASN (B) and pCTSbASN (C).

**Fig 2:**
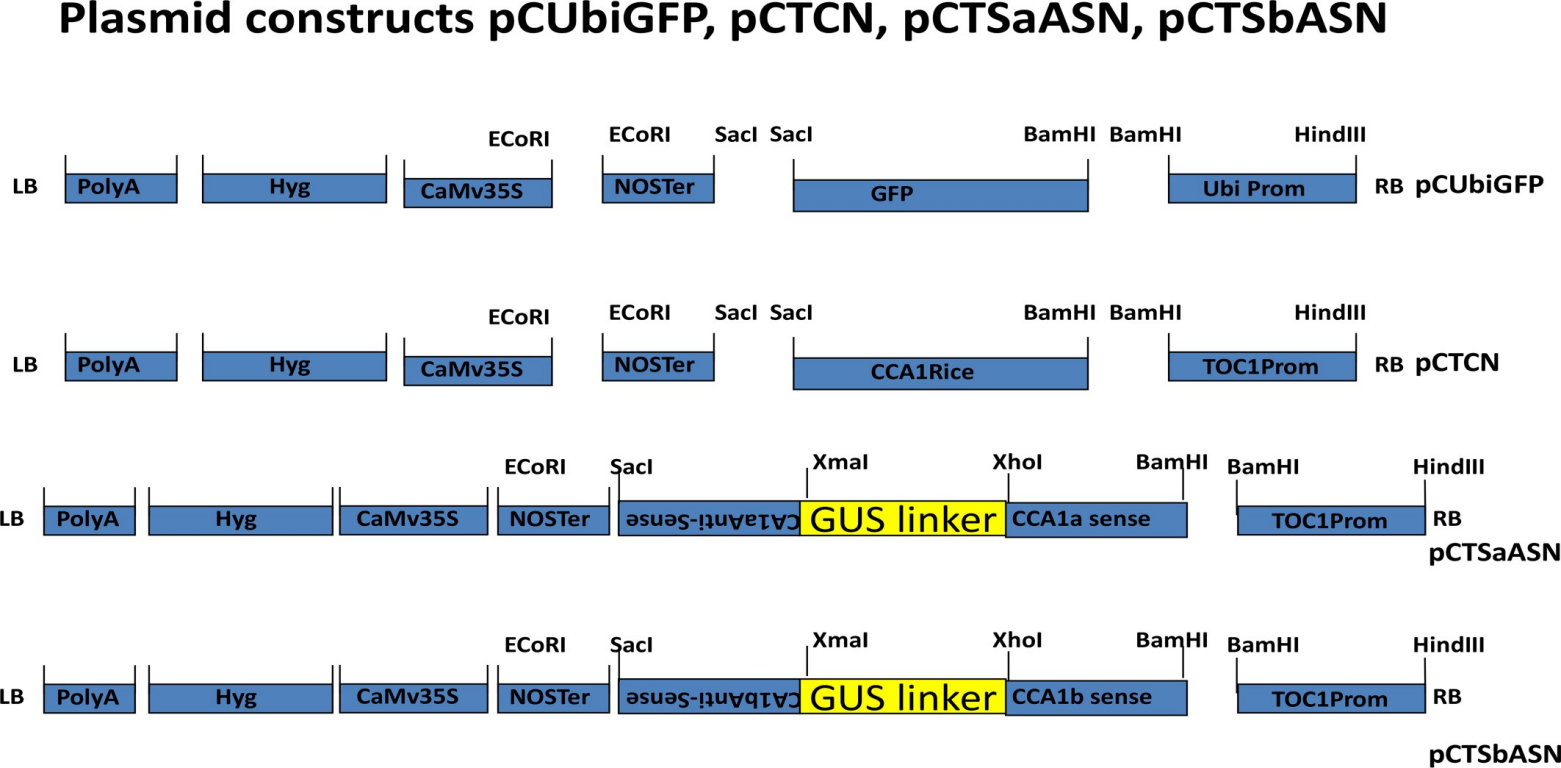
Plasmid constructs employed in the present investigation. The *gfp* gene under the control of Ubiquitin promoter (*pCUbiGFP*). One over expression cassette pCTCN designated as construct A having *TOC1*:*CCA1*:*NOS* was generated and two additional RNAi constructs, namely, *pCTSaASN (B)* and *pCTSbASN* (*C)* for repression of circadian clock genes have been made by cloning sense and antisense part of the *CCA1* gene (*a* & *b*) linked with GUS linker in pCAMBIA1300 under the control of *TOC1* promoter. Gene cassettes cloned in T-DNA region of binary vector pCAMBIA1300 LB- Left border, RB- Right Border, *CCA1*- Circadian Clock Associated, *TOC1* PRO- TIMING OF CAB EXPRESSION 1 PROMOTER, The *CCA1a* and *CCA1b* sense and antisense fragments for making the two RNAi constructs pCTSaASN and pCTSbASN were designed by choosing 400 bp of 5`region of *CCA1a* gene and 395 bp of 3`region of *CCA1b* gene, *CaMV35S*-Cauliflower Mosaic Virus 35 S Promoter, *NOS* Terminator- Nopaline synthase terminator.

Two colonies A1, A2, B1, B2, C1 and C2 were randomly picked from each of the three plates having constructs A, B and C mobilized in *Agrobacterium tumefaciens* and plasmid DNA was isolated using Qiagen mini prep kit. The presence of cloned gene constructs was confirmed by performing PCR amplification using 2μl of the isolated plasmid DNA of all the three constructs using primers for *TOC1* gene promoter. All the constructs showed the appearance of 1.35 kb *TOC1* band amplification as shown in Fig 1F. In addition, the purified plasmid DNA of all three constructs were sent for sequencing of *TOC1*, *CCA1* gene and were found to be correct and in the right orientation. Thus, one over expression cassette pCTCN (A) having *TOC1*:*CCA1*:*NOS* was generated and two additional RNAi constructs, namely, *pCTSaASN (B)* and *pCTSbASN* (*C)* for repression of circadian clock genes have been made by cloning sense and antisense part of the *CCA1* gene (*a* & *b*) linked with GUS linker in pCAMBIA1300 under the control of *TOC1* promoter as shown in Fig 2. Similar results of cloning and construction of RNAi vectors have been reported for *FLC1* gene in *Arabidopsis* [29], *GBNSS1* gene in potato [30], and *CCA1* gene in *Arabidopsis* [12].

### Callus induction, co-cultivation and *agrobacterium*-mediated genetic transformation

**Fig 3:**
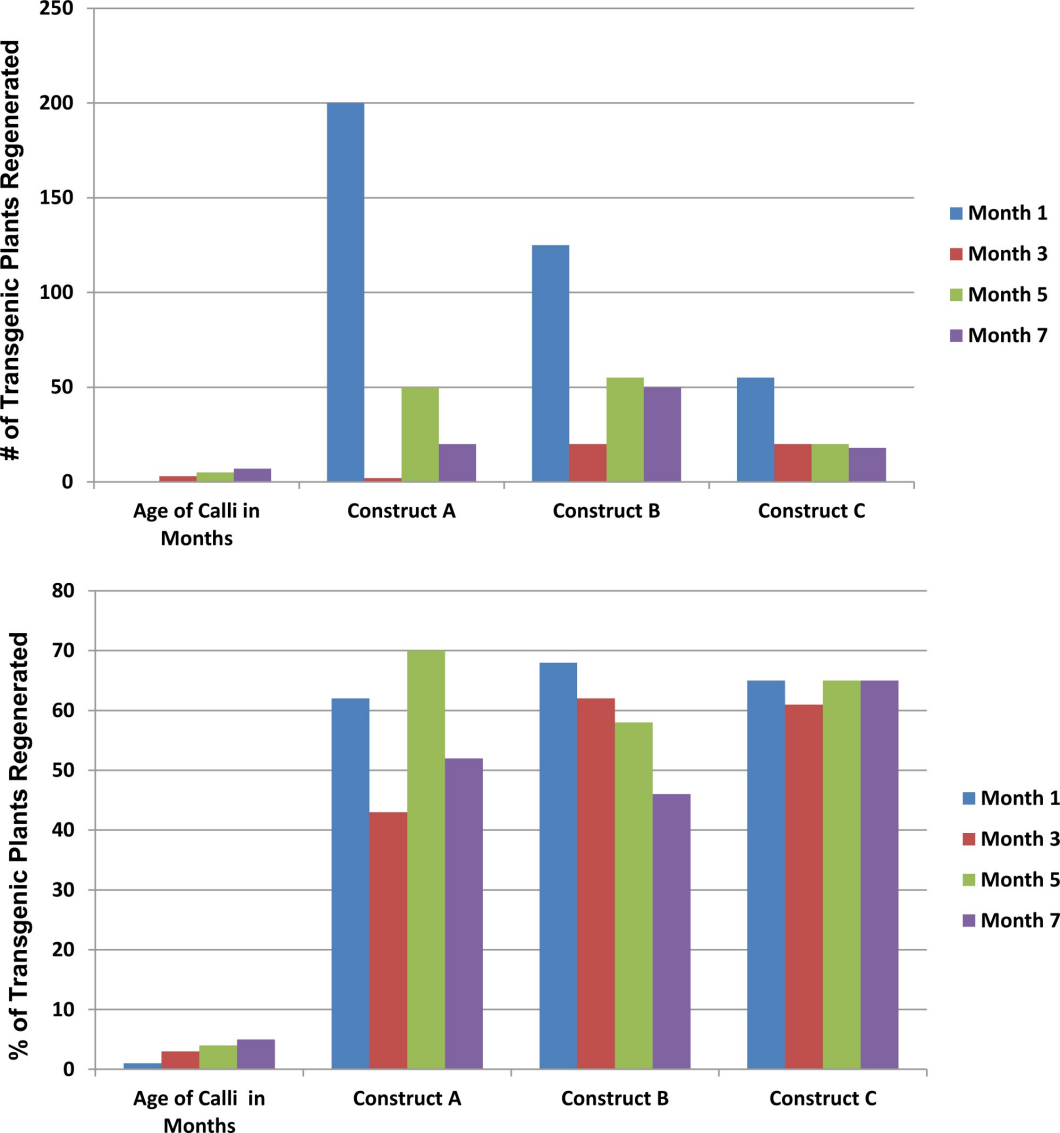
Callus induction, co-cultivation and *Agrobacterium*-mediated genetic transformation of *Japonica* rice variety Taipei 309. (A) and (B) High frequency plant regeneration from GFP expressing transformed calli. (C, D, E) High expression of GFP in the callus and roots of the transgenic plants; control roots did not show any *GFP* expression under confocal microscope (F, G) Creamy white to yellow nodular embryogenic calli proliferated well upon subculture, and 3 month old embryogenic calli (H) Selection and proliferation of hygromycin resistant calli on selection medium harboring constructs *A*, *B* and *C*. (I) The regenerated calli transferred on RM-I showing appearance of green spots after 3–4 weeks, produced more than one shoot derived from constructs A, B and C. (J) Regenerated transformed shoots were transferred to RM-II produced multiple shoots and well developed roots derived from constructs A, B and C. (K) The well developed putative transgenic plants derived from constructs A, B and C were transferred to plastic pots and maintained in Phytotron for acclimatization, hardening and further proliferation. (L) The transgenic plants of *GFP*, A, B, C construct and wild type were maintained in Phytotron for panicle formation, seed setting and grown till maturity.

*Agrobacterium*-mediated genetic transformation of Japonica rice variety Taipei 309 using scutellum derived calli was optimized having *gfp* gene under the control of ubiquitin promoter (*pCUbiGFP*) which resulted in high regeneration frequency on hyg containing medium as shown in Fig 3A and 3B and also high level of *gfp* expression in roots of transformed plants, whereas, control roots did not show any *GFP* expression under confocal microscope as shown in Fig 3C and 3D. Calli, regenerating shoot buds and roots growing on hyg exhibited high level of gfp expression whereas, control roots did not show any *gfp* expression as shown in Fig 3E.

Germination of seeds occurred 72h after transferring on callus induction medium and the creamy white to yellow nodular embryogenic calli proliferated well upon subculture as shown in Fig 3F and 3 month old embryogenic calli (Fig 3G). Scutellum derived calli of 1, 3, 4 and 5 months old were co-cultivated with *Agrobacterium* harboring gene constructs A, B and C. The hyg resistant calli were recovered on selection medium. The hygromycin restricted the growth of non-transformed calli, which turned brown and the transformed calli showed active cell proliferation derived from constructs A, B and C as shown in Fig 3H. The *hyg* resistant calli were transferred on to RM-I. The regenerated calli transferred on RM-I showed green spots after 3–4 weeks, produced more than one shoot derived from constructs A, B and C as shown in Fig 3I. The shoot-regenerating calli were transferred to RM-II and the remaining calli were kept on fresh RM-I for another 2 weeks to check further regeneration potential. The plantlets on RM-II medium have produced multiple shoots, derived from constructs A, B and C as shown in (Fig 3J) and initiated root formation. The well developed putative transgenic plants derived from constructs A, B and C were transferred to plastic pots and maintained in Phytotron for acclimatization, hardening and further proliferation (Fig 3K). The transgenic plants of *GFP*, A, B, C construct and wild type were maintained in Phytotron for panicle formation, seed setting and grown till maturity (Fig 3L). It was observed that the responses of 1 month old callus were better for transformation and plant regeneration than 3, 4 and 5 month old callus as well as inclusion of BAP in callus induction medium. Similar results were reported for Japonica rice variety Taipei 309 and perennial rye grass [22]. Similarly, high frequency plant regeneration from embryogenic calli of Bermuda grass by inclusion of BAP in callus induction medium was reported [31].

**Fig 4:**
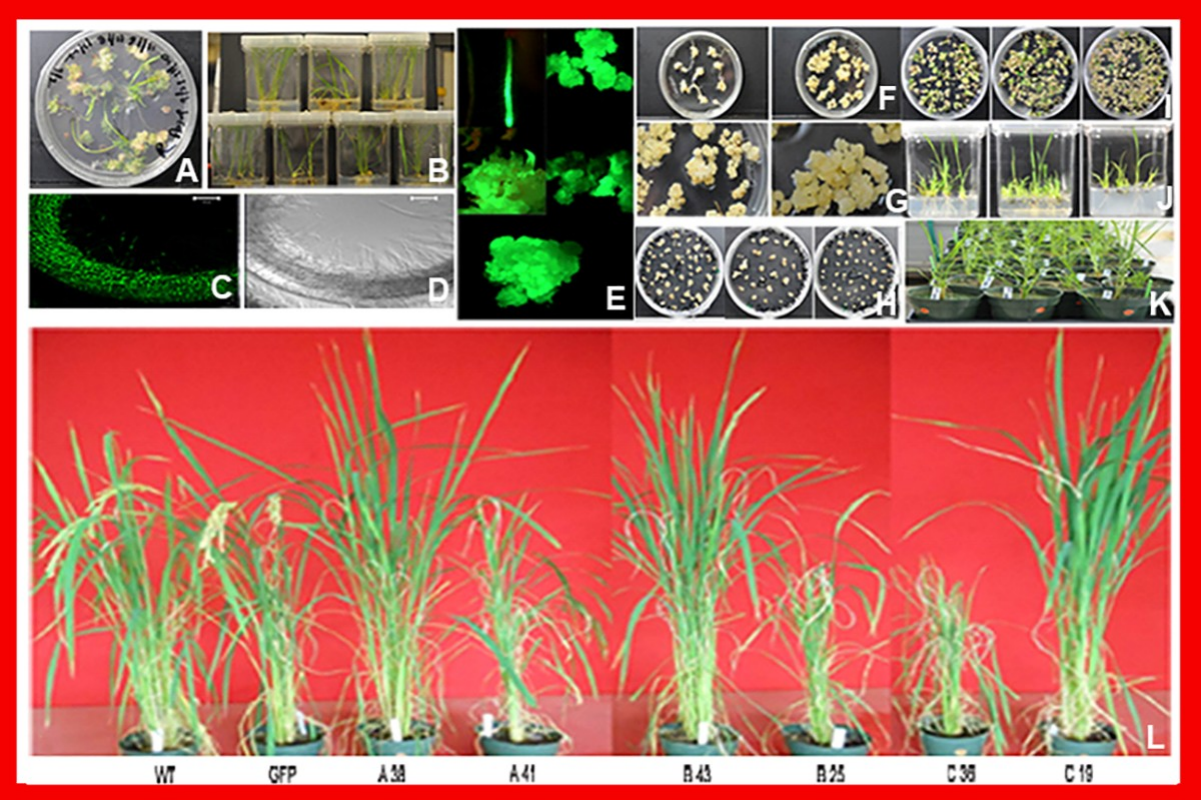
**Transgenic plants (T0) regenerated from *Agrobacterium*–mediated genetic transformation of rice calli having 1, 3, 4 & 5 month old using circadian clock gene constructs A, B, & C.** (A) Number of transgenic plants regenerated. (B) Percentage of transgenic plants regenerated.

Maximum number of transgenic plants regenerated from *Agrobacterium*-mediated genetic transformation of 1 month old rice calli (184 plants) while the lowest number of transgenic plants regenerated from *Agrobacterium*-mediated genetic transformation of 3 month old rice calli (34 plants) as shown in Fig 4A total of 632 transgenic plants were generated from *Agrobacterium*-mediated genetic transformation of rice calli having 1, 3, 4 and 5 month old using circadian clock gene constructs A, B and C. The over expressing construct A generated 270 plants and the RNAi construct B produced 258 transgenic plants, whereas, RNAi construct C produced 104 transgenic plants as shown in Fig 4. The regeneration frequency of transformed shoots varied from 47–70% depending upon the age of the calli at the time of co-cultivation varying from 1, 3, 4 and 5 month old (Fig 4). Therefore, plant regeneration frequency decreases with increase in the age of calli. Similar results were reported in which the response of 2–3 month old rice callus was better for transformation and plant regeneration than 4–6 month-old callus [22]. A total of 90 transgenic plants comprising 30 plants each (independently transformed lines) for each of the constructs, namely, A, B & C and six control plants comprising of three GFP transgenic plants and three wild type plants were transferred to 6 inch pots in peat-lite/sand. In the present study MS based infection medium with 200 μM Acetosyringone has been successfully used for 15 min of *Agro*-infection inclusive of 3 min of heat shock at 42°C. MS based *Agro*-infection medium has been successfully reported earlier [22, 32–35]. It is widely believed that transformation normally takes place within actively dividing cells and MS medium is better for plant cell growth compared to YEP medium. Similarly, Han *et al*. [35] also reported improved transformation efficiency in *Artemisia annua* L. while using MS as an infection medium rather than the LB medium.

### PCR analysis and Southern analysis of T0, T1 and T2 transgenic plants

**Fig 5:**
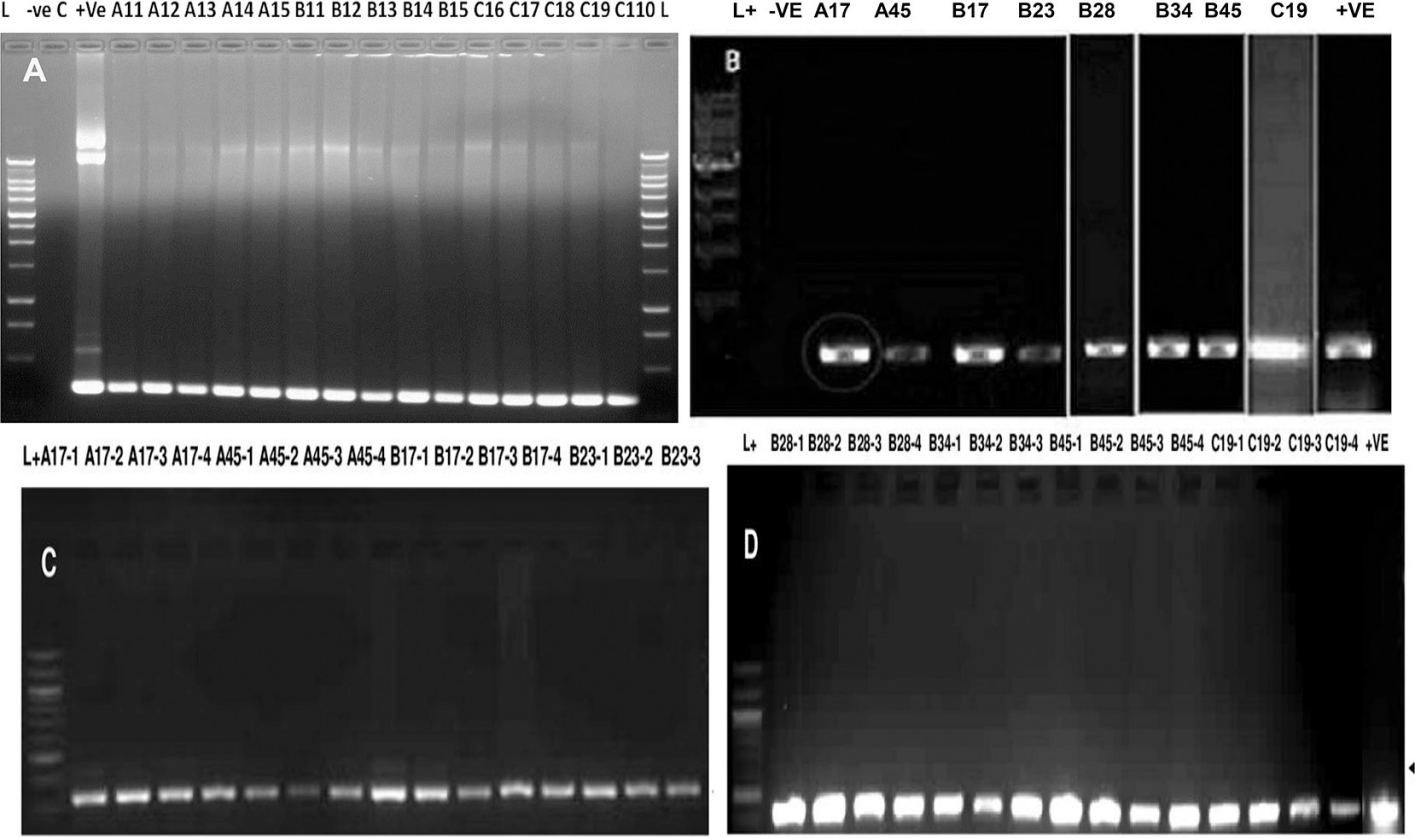
PCR analysis of T0, T1 and T2 transgenic plants for the presence of the *hyg* gene. Arrowhead indicates the 324 bp band corresponding to the *hyg* gene of the T-DNA. The pCAMBIA1300 served as a Positive Control (+ve) and non-transgenic Taipei 309 served as a Negative Control (–ve). (A) PCR Analysis of T0 Transgenic Lane 1 L—1 kb ladder, lane 2- (–ve control, lane 3 +ve control, lanes 4–8—putative transgenic plants of construct A, lanes 9–13- putative transgenic plants of construct B, lanes 14–18 putative transgenic plants of construct C, lane 19–1 kb ladder. (B) PCR Analysis of T1 Transgenic Plants. Lane 1- L—1 kb ladder, lane 2 –ve control, lanes 3–4 putative transgenic plants of construct A, lanes 5–9- putative transgenic plants of construct B, lanes 10—putative transgenic plants of construct C, lane 11 +ve control. (C) PCR Analysis of T2 Transgenic Plants. Lane 1- L—1 kb ladder, lane 2–9 putative transgenic plants of construct A, lanes 10–16 putative transgenic plants of construct B. (D) PCR Analysis of T2Transgenic Plants. Lane 1- L—1 kb ladder, lanes 2–12 putative transgenic plants of construct B, lanes 13–16 putative transgenic plants of construct C, lane 17 +ve control. The gel images for Fig 5A, 5C and 5D is original. The gel images for Fig 5B have been cropped; full length gel pictures have been shown as S7 Fig.

Before performing the expression analysis of *CCA1* gene in independent T0 transgenic lines, integration of the selectable marker was confirmed by PCR amplification of *hyg* gene in putative transgenic lines of *Japonica* rice variety Taipei 309 as shown in Fig 5A. The wild type served as a negative control without any amplification, whereas, positive control (pCAMBIA1300), and all the putative transgenic plants of construct A, B and C showed amplification of 324 bp fragment of *hyg* gene indicating, thereby, that T-DNA had been inserted into the genome of all regenerated putative transgenic plants tested. Similarly, randomly selected T1 and T2 progeny plants of constructs A, B and C also showed 324bp fragments of hygromycin selectable marker gene indicating T-DNA has been successfully integrated and inherited in T1and T2 progeny plants as shown in Fig 5B, 5C and 5D.

**Fig 6:**
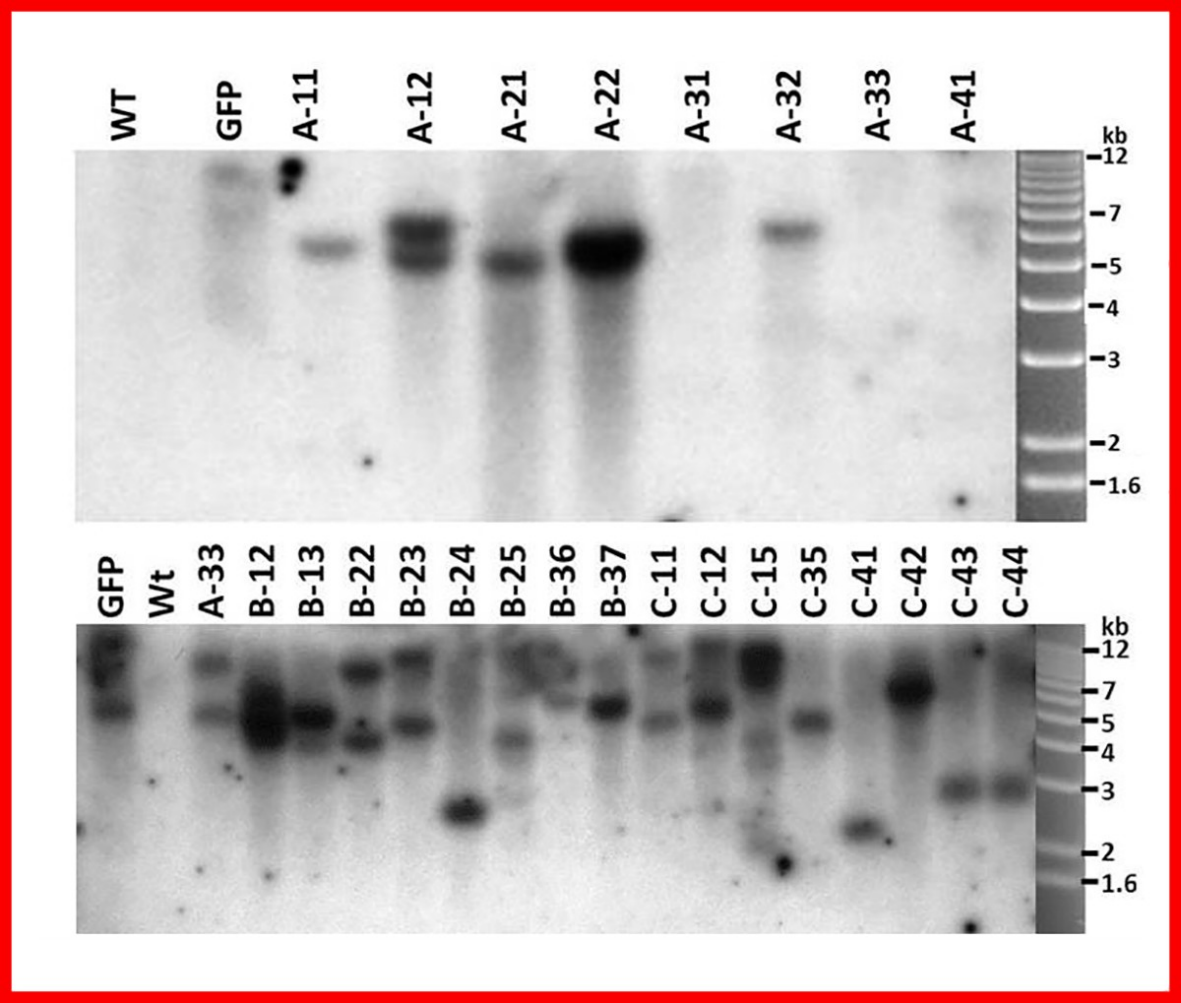
**Southern blot analysis of T0 transgenic plants derived from wild type, *gfp* gene containing transgenic plants and twenty five transgenic plants derived from constructs A, B and C.** The genomic DNA was digested with *HindIII* and probed with *hyg* gene.

Southern blot analysis was performed to confirm the integration of the desired genes into the rice genome of T0 transgenic rice plants. The wild type served as a negative control without any appearance of band, whereas, *gfp* gene expressing transgenic plants, and putative transgenic plants of construct A, B and C showed Southern positive bands. Transgenic lines A-11, A-22 A-32, B-13, B-24, B-37, C-35, C-41, C-43 contained single band, indicating single copy (Fig 6) or more than one bands in transgenic lines A-12, A-33, B-12, B-22, B-23, B-25, B-36, B-37 indicating multiple copies (Fig 6). Thus, stable integration of T-DNA in the T0 transgenic plants derived from construct A, B and C was confirmed as shown in Fig 6. Similarly; molecular analysis were carried out by most of the workers to check the integration pattern and copy number by Southern blot analysis [36–38], reported one to two copy number of the transgene in T0 plants and one and three copy number integration of transgene in T1 independent transformants using Southern blot analysis.

### Quantitative real time expression analysis of *CCA1* gene and morphological data of T0, T1 and T2 transgenic progeny plants

**Fig 7:**
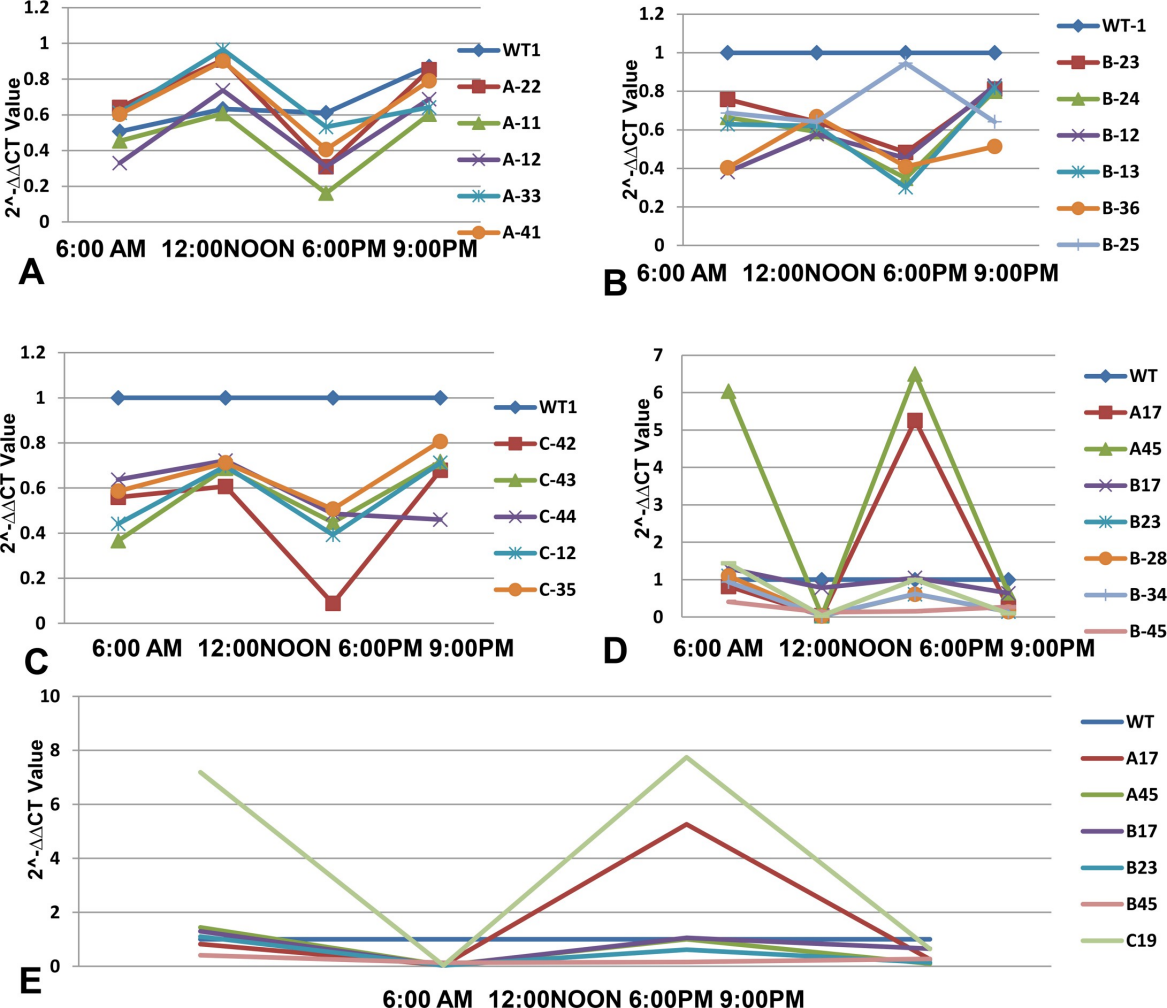
The qRT-PCR expression analysis of *CCA1* gene in T0, T1 and T2 transgenic rice plants at different time points; 6:00AM, 12:00 Noon, 6:00 PM and 9:00 PM. The relative fold change expression of *CCA1* gene was monitored as per 2^-ΔΔCT^ method by taking wild type as a calibrator. (A, B, C) *CCA1* gene expression in T0 derived transgenic plants of construct A, B and C (D) *CCA1* gene expression in T1 derived transgenic plants of construct A, B and C (E) *CCA1* gene expression in T2 derived transgenic plants of construct A, B and C.

**Fig 8:**
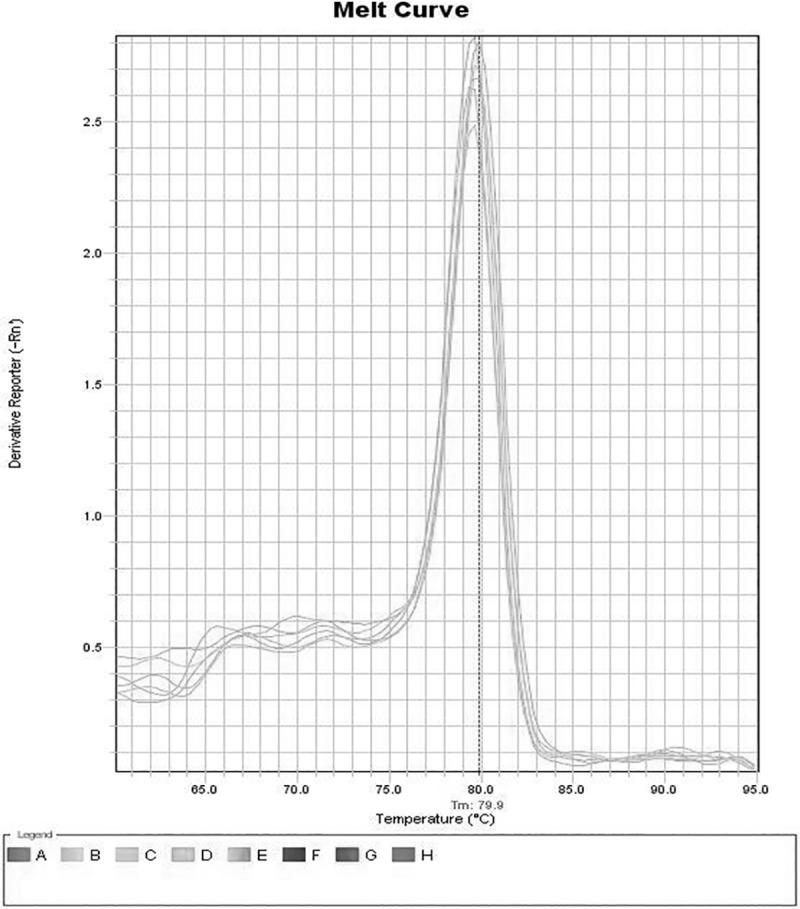
Melt curve of 18 *S rRNA* primers employed as an internal control (GenBank Accession No. AF156675) for qRT-PCR analysis of various T1 transgenic progeny plants of rice.

**Fig 9:**
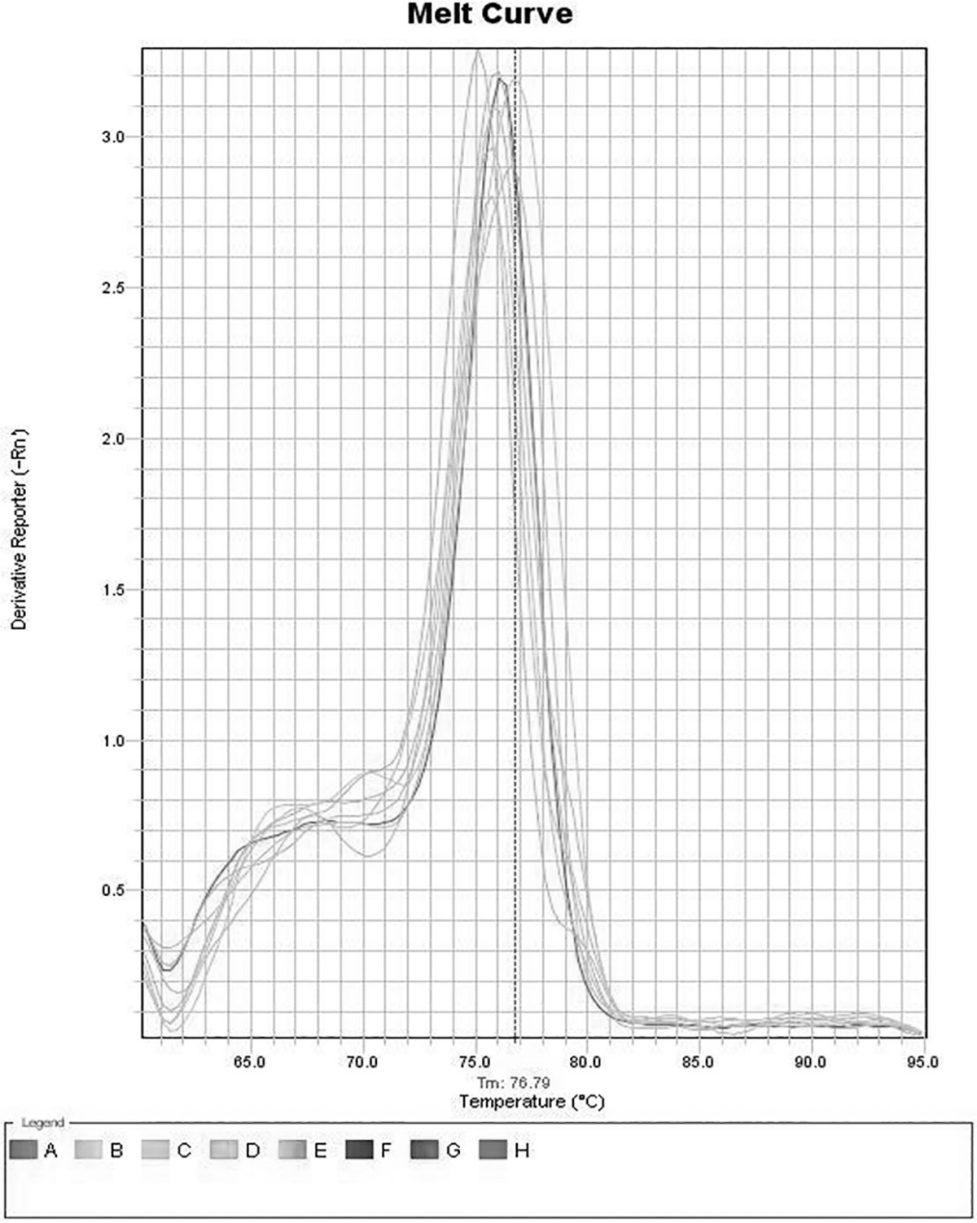
Melt curve of *CCA1* gene-specific primers employed in qRT-PCR analysis of various T1 transgenic progeny plants of rice.

Quantitative real time PCR of various transgenic T0 lines, T1 lines and T2 progeny lines was carried out for ascertaining the *CCA1* gene expression for constructs A, B and C. The 2^-ΔΔCT^ method was used to calculate relative changes in expression of *CCA1* gene. Amplicon abundance was monitored in real-time by measuring SYBR Green fluorescence. The level of *CCA1* gene transcript in T0, T1 and T2 plants was normalized with reference to *18S rRNA* taken as an internal control as shown in Fig 7. The specificity of primers employed in the present investigation for 18 *S rRNA* as an internal control and the gene of interest *CCA1* in T1 transgenic progeny plants of rice in qRT-PCR analysis as Melt curve is exhibited in Figs 8 and 9, respectively, similar results were obtained in T2 transgenic progeny plants. The expression profile of the T0, T1 and T2 transgenic lines was made in comparison to the wild type (WT) non transformed plant, which was taken as the calibrator. The expression profile of T0 transgenic lines showed that all the transgenic lines derived from construct A, B and C showed highest *CCA1* gene expression at 12:00 Noon and lowest level of *CCA1* gene expression at 6:00 PM as shown in Fig 7A to 7C. All the T0 transgenic plants derived from RNAi constructs B and C, namely, B-12, B-13, B-23, B-24, B-36, C-12, C-35, C-42, C-43 and C-44 consistently exhibited repression of *CCA1* gene at 6:00PM, whereas, B-25 exhibited highest expression at 6:00PM as compared to construct A derived over-expressing T0 transgenic plants A-11, A-12, A-22, A-33 and A-41which is correlated with *CCA1* over expression as shown in Fig 7A, 7B and 7C.

Similarly, the *CCA1* gene expression profile of T1 transgenic lines showed that the transgenic lines derived from construct A, A-17, A-45 showed high level of *CCA1*gene expression at 6:00 AM and lowest level of *CCA1* gene expression at 12:00 Noon, which again increased by 6 folds and 1 fold at 6:00 PM followed by decrease at 9:00 PM in a rhythmic manner as shown in Fig 7D. Similarly, T1and T2 transgenic plants derived from constructs B and C, namely, B-17, B-23, B-28, B-34, B-45, and C-19 showed lowest *CCA1* gene expression at 12:00 Noon and highest level of *CCA1* gene expression at 6:00 AM except B-17 which again show high expression of *CCA1* gene expression at 6:00 PM as shown in Fig 7D and Fig 7E. Similar results of robust expression and association of *ZmCCA1* with circadian clock in maize was reported [14] and *CCA1* gene expression peaking at dawn, which began to decrease rapidly in *Arabidopsis thaliana* was reported [12].

The results of the present study are in conformation with the findings of Ni *et al*. [12] that repression of *CCA1* gene promotes growth, biomass, yield, sugar and starch content and chlorophyll content while the over-expression of *CCA1* would result in decreased growth vigor in diploids of *Arabidopsis*. The *CCA1* gene expression of T1and T2 progeny lines in the present investigation is highest at dawn (6:00 AM), lowest at 12:00 Noon, again increases until dusk at 6:00 PM and then starts decreasing at 9:00 PM in a rhythmic pattern. At the same time construct A derived transgenic T1 and T2 plants exhibited several fold higher expression of *CCA1* gene at dusk, whereas, construct B and C derived transgenic T1and T2 plants exhibited several folds lower expression of *CCA1* gene at 12:00 Noon. Likewise, Ni *et al*. [12] observed that the *CCA1* gene expression peaked at dawn (Zeitgeber time 0, ZT0), decreased 6 h after dawn (ZT6), and continued declining until dusk (ZT15). They reported that at ZT6 (noon), *CCA1* and *LHY* were repressed, 2-fold, whereas *TOC1* was up-regulated, 2-fold in the F1 hybrids relative to the parents. They also reported that the *CCA1* gene expression *TOC1*::*CCA1*(RNAi) transgenic *Arabidopsis* plants, *CCA1* messenger RNA and protein amounts were down-regulated 2–10-fold and 1.4–3-fold, respectively, and *CCA1* gene repression results in increased chlorophyll synthesis, starch metabolism and growth vigour and this data is in agreement to the data obtained in the present investigation. Similarly, Wang *et al*. [14] characterized *CCA1* gene of maize (*ZmCCA1*), and reported rhythmic expression of *ZmCCA1* in leaves and stem apex meristems under long day and short day conditions, and its peak gene expression appeared during the morning. They proposed that *ZmCCA1* may be a core component of the circadian clock in maize.

Similarly, quantitative-PCR techniques are used extensively to assay and characterize clock gene expression, most notably *CCA1* and *LHY*, directly over a sampling time-course in circadian studies in conjunction with high-throughput techniques [1, 2, 14, 39]. Wang and Tobin quantified *CCA1* RNA by competitive RT-PCR in transgenic *Arabidopsis* plants and showed that constitutive expression of *CCA1* results in longer hypocotyls and substantially delayed flowering [2]. Similarly, Lee *et al*. [40] reported a rhythmic expression of MYB96- a transcription factor that is connected with the clock oscillator that peaked around dusk using qRT-PCR analysis in *Arabidopsis*.

Morphological characteristics of T1 transgenic progeny plants at different developmental stages from germination of seeds to flowering and seed setting are shown in S4 Fig. Transgenic T0 and T1 seeds from three constructs A, B and C were germinated in plastic pots in Transgenic Green House to obtain T1and T2 progeny plants derived from constructs A, B and C on mixture of soil, vermiculite after one week as shown in S4A Fig. The transgenic plants proliferated well after 2–3 weeks and produced multiple shoots as shown in S4B Fig. The transgenic plants derived from RNAi Constructs B and C; B-17 and C-19 showed better growth, number of tillers/panicles as compared to plants derived from Construct A; A-17 as shown in S4C Fig. The flowering stage (immature panicles stage or milky stage) of transgenic T1 progeny plants derived from constructs A, B and C are shown in S4D Fig. The well developed transgenic T1and T2 progeny plants were transferred to plastic pots containing soil mixture and maintained in Transgenic Green House for further proliferation, flowering, panicle formation and seed setting and grown to maturity till harvesting as shown in S4E Fig. Similar morphological characteristics of T2 transgenic progeny plants at different developmental stages from germination of seeds to flowering and seed setting are shown in S5 Fig. T1 and T2 transgenic progeny plants were further analyzed for their morphological traits and growth characteristics such as plant height, numbers of tillers/panicle, yield, thousand seed weight, seed length, seed width and chlorophyll content at different point of time and subjected to statistical analysis using Student’s *t*-test.

**Fig 10:**
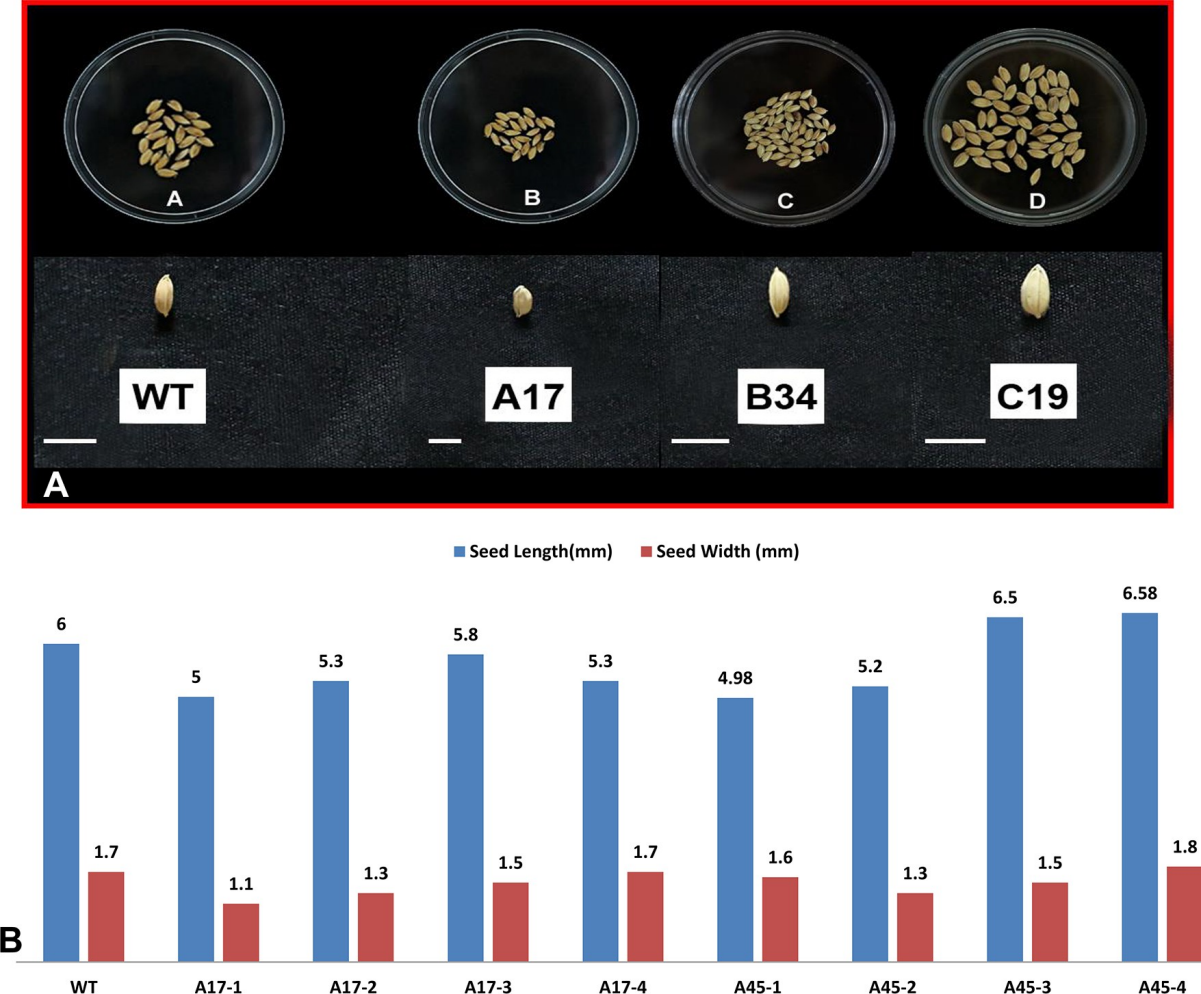
**Morphological appearance and comparison of seed size of T1 transgenic progeny plants harboring gene constructs *A*, *B* and *C* and that of wild type (WT).** Bar size is in mm. (A) morphological appearance of T1 seeds. Bar size depicting seed length for WT-6.75mm, Construct A-5.5mm, Construct B-7.02mm and Construct C-7.43mm. (B) Seed length and width of WT and various lines derived from construct A, B and C are shown graphically.

**Fig 11:**
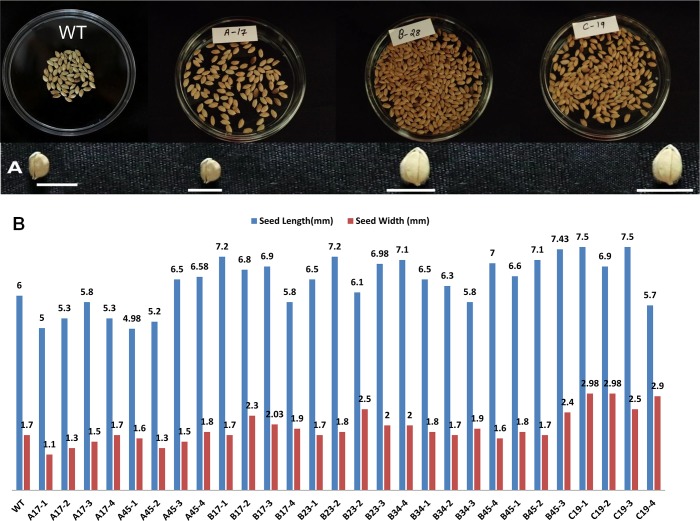
**Morphological appearance and comparison of seed size of T2 transgenic progeny plants harboring gene constructs *A*, *B* and *C* and that of wild type (WT).** Bar size is in mm. (A) morphological appearance of T2 seeds. Bar size depicting seed length for WT is 6.75mm, Construct A- 5.5mm, Construct B-7.02mm and Construct C-7.5mm. (B) Seed length and width of WT and various lines derived from construct A, B and C are shown graphically.

Characterization of T0 transgenic lines of construct A, B and C did not show any considerable difference in any of the morphological traits over the wild type and in student’s *t*-test was also not found statistically significant as shown in Table 2. While, T1 transgenic progeny plants derived from constructs B and C showed increased number of tillers/panicles, improved plant condition, better seed yield, increased thousand seed weight, slightly increased seed size as compared to wild type plants, at the same time T1and T2 transgenic progeny plants derived from constructs A exhibited reduced number of tillers/panicles, reduced yield, reduced thousand seed weight and decreased seed size as compared to wild type plants as shown in Tables 3 and 4 and Figs 10 and 11. The T1transgenic progeny plants derived from constructs A, namely, A-17 and A-45 exhibited reduced number of tillers/panicles (6–7), reduced thousand seed weight (16.6–15.5g), decreased seed length (5.5–5.4mm) and decreased seed width (1.3–1.75mm) as compared to wild type plants as shown in Table 3, S1 Table and Fig 10. The T2 transgenic progeny plants derived from constructs A, namely, A-17-1 to A17-4 and A-45-1 to A-45-4 exhibited reduced number of tillers/panicles (5–9), reduced thousand seed weight (10.1–14.5g), decreased seed length (4.98–6.58mm) and decreased seed width (1.1–1.8mm) as compared to wild type plants as shown in Table 4, S2 Table and Fig 11. On the contrary, T1 transgenic progeny plants derived from constructs B and C showed increased number of tillers/panicles (8–16), improved plant condition (3–4), better seed yield (3.95–23.01g), increased thousand seed weight (16.8–28.5g), slightly increased seed length (6.75–7.43 mm), and increased seed width (1.8–2.18 mm) as compared to wild type plants as shown in Table 3, S1 Table and Fig 10. Similarly, T2 transgenic progeny plants derived from constructs B and C showed increased number of tillers/panicles (8–19), improved plant condition (3–4), better seed yield (6.5–28.9g), increased thousand seed weight (16.9–29.03g), slightly increased seed length (6.7–7.5 mm) and increased seed width (1.75–2.98 mm) as compared to wild type plants as shown in Table 4, S2 Table and Fig 11.The *P* value in all the above cases was found to be less than 0.05 so the differences in morphological characters were found to be statistically significant as shown in Tables 3 and 4 in majority of the transgenic lines derived from B and C constructs of T1 and T2 progeny plants.

**Table 2:** Morphological characterization of T0 transgenic progeny plants.

T0 Plants	Plant Height (cm)	No. of Tillers/Panicle	Yield (g)
WT1	109	15	4.1
A-11	114	31	1.9
A-12	114	22	10.1
A-22	139	3	3.3
A-33	107	9	2.0
A-41	95	13	0.1
B-12	114	18	3.4
B-13	106	14	5.1
B-23	108	23	6.7
B-24	108	9	5.5
B-25	102	12	-
B-36	104	14	0.8
C-12	105	16	8.1
C-35	88	14	3.2
C-42	121	20	18.6
C43	111	20	2.7
C44	92	16	-

**Table 3:** Morphological characterization of T1 transgenic progeny plants with statistical analysis (student’s t-test).

Plants (T1 Progeny)	Plant Height (cm)	P value (<0.05)	No. Of Tillers/ Panicle	P value (<0.05)	Yield (g)	P value (<0.05)	1000 seed weight (g)	P value (<0.05)	Seed length (mm)	P value (<0.05)	Seed Width (mm)	P value (<0.05)	Chlorophyll content mgg^-1^	P value (<0.05)
WT	120	-	8	-	3.95	0.006	16.8		6.75		1.8	0.01	0.0392	
A-17	110	0.019	**6**	-	**4.84**	0.0005	**16.6**	0.02	**5.5**	0.0015	**1.3**	0.002	0.0351	-
A-45	115	0.012	**7**	-	**4.47**	0.004	**15.5**	0.035	**5.4**	0.031	**1.75**	0.013	0.0381	-
B-17	120	0.0032	**9**	0.0063	**8.95**	0.0006	21.7	0.00054	7.02	0.0063	2.01	0.011	0.0432	-
B-23	110	0.017	11	0.0053	11.70	0.0005	21.5	0.0045	**6.98**	0.0053	**1.98**	0.002	0.0398	-
B-28	120	0.021	10	0.024	10.08	0.00041	**21.2**	0.015	7.02	0.024	2	0.0021	0.0415	-
B-34	120	0.031	13	0.035	9.89	0.0004	22.0	0.017	6.98	0.035	2.03	0.0023	0.0385	-
B-45	120	0.028	14	0.023	17.01	00003	22.3	0.034	7.01	0.023	2.05	0.0023	0.0452	-
C-19	145	0.0018	**16**	0.004	**23.01**	0.0001	**28.5**	0.0005	**7.43**	-	**2.18**	0.002	0.0492	0.00065

**Note:** The data was subjected to statistical analysis using Student’s *t*-test. All the morphological data is presented as the mean ± SE and *P* value to compare the obtained parameters from transgenic lines (TG) and wild type plant (WT) and a *P* value of **<**0.05, was considered to be statistically significant. And sign dash–shows no significant difference was found. Highest and lowest values are in bold for Construct A and Construct B & C.

**Table 4:** Morphological characterization of T2 transgenic progeny plants with statistical analysis (student’s t-test).

Plants (T2 Progeny)	Plant Height (cm)	P value (<0.05)	No. Of Tillers/ Panicle	P value (<0.05)	Yield (g)	P value (<0.05)	1000 seed weight (g)	P value (<0.05)	Seed length (mm)	P value (<0.05)	Seed Width (mm)	P value (<0.05)	Chlorophyll content mgg^-1^	P value (<0.05)
WT	120		8		6.5	0.01	16.9	-	6.7	-	1.75	0.01	0.045	-
A-17-1	**100**	0.015	**5**	-	8.03	0.004	13.5	0.001	**5**	-	**1.1**	0.002	0.0351	0.01
A-17-2	110	0.011	6	-	**2.17**	0.05	11.3	0.02	5.3	-	1.3	-	0.0381	0.012
A-17-3	105	0.01	**7**	-	**8.34**	0.0001	**14.5**	0.035	5.8	-	1.5	-	0.036	0.017
A-17-4	100	0.014	8	**-**	3.14	0.04	12.6	0.00054	5.3	-	1.7	-	0.037	-
A-45-1	110	0.017	7	-	3.5	0.05	13.8	0.0045	4.98	-	1.6	0.0021	0.029	0.013
A-45-2	115	0.019	**9**	-	6.65	0.0005	**10.1**	0.015	5.2	-	1.3	0.0020	0.028	0.012
A-45-3	**120**	0.012	7	-	5	0.043	11.5	0.017	6.5	-	1.5	0.023	0.035	0.017
A-45-4	110	0.019	8	-	3.66	0.03	13.07	0.034	**6.58**	0.01	**1.8**	0.001	0.0383	0.01
B-17-1	**125**	0.028	9	0.0061	12.7	0.0005	18.7	0.0005	7.2	0.0063	**1.7**	0.013	0.0432	0.014
B-17-2	130	0.037	9	0.0045	**4.98**	0.041	**15.6**	0.01	6.8	0.0043	2.3	0.019	0.0398	0.015
B-17-3	135	0.0032	9	0.024	5.9	0.033	17.9	0.0035	6.9	0.024	2.03	0.002	0.0415	0.00065
B-17-4	130	0.017	**8**	0.035	5.7	0.032	16.8	0.00054	5.8	0.031	1.9	0.001	0.0385	0.012
B23-1	128	0.021	11	0.033	20.1	0.0001	19.8	0.0045	6.5	0.032	1.7	0.0023	0.0452	0.001
B-23-2	135	0.031	12	0.004	14.7	0.0032	22.2	0.015	7.2	0.003	1.8	0.005	0.04164	0.018
B-23-3	130	0.028	14	0.012	10.5	0.0062	20.1	0.017	6.1	0.024	2.5	0.002	0.048	0.0035
B-23-4	132	0.022	12	0.001	14.7	0.0003	19.9	0.034	6.98	0.042	2	0.019	0.043	0.00054
B-28-1	130	0.025	11	0.009	14.7	0.0003	17.6	0.027	6.88	0.039	1.94	0.004	0.0445	0.013
B-28-2	135	0.032	10	0.001	8.83	0.0053	20.3	0.0012	7.01	0.041	2.03	0.013	0.0382	0.00065
B-28-3	134	0.031	10	0.001	16.21	0.00041	15.8	0.025	6.75	0.039	1.96	0.039	0.0419	0.015
B-28-4	136	0.0033	11	0.009	8.46	0.0051	19.3	0.0018	6.92	0.041	2.14	0.05	0.045	0.041
B-34-4	130	0.0221	10	0.018	14.6	0.0001	18.06	0.0005	7.1	0.023	2	0.013	0.042	0.0045
B-34-1	133	0.0032	9	0.023	16.2	0.006	17.8	0.034	6.5	0.01	1.8	0.011	0.048	0.01
B-34-2	135	0.017	10	0.036	8.86	0.025	16.5	0.0005	6.3	0.023	1.7	0.002	0.038	0.014
B-34-3	145	0.021	12	0.021	14.4	0.004	18.9	0.001	**5.8**	0.033	1.9	0.0021	0.041	0.017
B-45-4	138	0.031	11	0.004	23.9	0.0001	20.6	0.002	7	0.0034	1.6	0.0023	0.0421	0.023
B-45-1	137	0.028	11	0.028	24.6	0.0002	22.2	0.0035	6.6	0.001	1.8	0.0023	0.0398	0.002
B-45-2	135	0.020	14	0.024	17.86	0.0004	21.9	0.00054	7.1	0.0012	1.7	0.002	0.025	0.035
B45-3	130	0.032	15	0.033	14.9	0.0004	18.7	0.0045	7.43	0.017	2.4	0.013	0.028	0.0054
C-19-1	145	0.025	15	0.023	19.86	00003	28.3	0.0015	**7.5**	0.0063	2.98	0.011	0.045	0.0045
C-19-2	**150**	0.0031	17	0.002	25.8	0.0001	28.5	0.0017	6.9	0.0053	**2.98**	0.002	0.0498	0.001
C-19-3	148	0.015	18	0.030	**28.9**	0.0011	**29.03**	0.034	7.5	0.024	2.5	0.0021	0.0492	0.001
C-19-4	148	0.01	**19**	0.01	19.86	0.0016	25.6	0.0003	7.5	0.0025	2.9	0.0023	0.0487	

**Note:** The data was subjected to statistical analysis using Student’s *t*-test. All the morphological data is presented as the mean ± SE and *P* value to compare the obtained parameters from transgenic lines (TG) and wild type plant (WT) and a *P* value of **<**0.05, was considered to be statistically significant. And sign dash–shows no significant difference was found. Highest and lowest values are in bold for Construct A and Construct B & C.

Increase of seed size in wild type Japonica rice variety Nipponbare has been reported by Liu *et al*. [41], GW5 protein is expressed in various rice organs and acts in the brassinosteroid signaling pathway regulating grain width and weight. Similarly, in wild type tropical Japonica rice, *GLW7* encoding the plant-specific transcription factor OsSPL13 positively regulates cell size in the grain hull, resulting in enhanced rice grain length and yield has been reported [42]. Recently, over-expression of *OsEXPA10* gene in rice enhancing growth, which lead to increased susceptibility to BPH (Brown Plant Hopper) being one of the nastiest insect pests of rice and blast attack has been reported by Tan *et al*. [43]. Repression *OsEXPA10* gene reduced morphological characters but improved resistance to BPH and blast attack. The results of present investigation for over-expression of *CCA1* gene in T1 and T2 transgenic progeny plants resulting in detrimental effect on morphological attributes, whereas, repression of *CCA1* gene by RNAi constructs in T1and T2 transgenic plants resulted in improved morphological characteristics are in conformity of data presented [12, 14] and for increased chlorophyll synthesis, starch metabolism and growth vigour [40] by RNAi constructs harboring *CCA1* gene in transgenic *Arabidopsis thaliana* plants. Recently, mathematical models and experimental data in *Arabidopsis thaliana* showed that Myb transcription factors CCA1/LHY proteins serve as a repressor and RVE8 acts as an activator of gene expression even though both of them bind to the same cis-element [44]. They have shown that multiple feedback loops of the plant clock genes guarantee rhythmicity under unfavorable environmental cues. A variable response of gene expression between scion and rootstock upon homo grafting of *Arabidopsis thaliana* in various organs was reported from my laboratory [45]. The flower buds of scion showed over-representation of the transcription factor genes, such as Homeobox, MYB and NAC. Differential transcription of genes related to gibberellic acid, ethylene and other stimuli was observed between scion and rootstock.

Our results of gene silencing by RNAi vectors are in conformity to the data reported for *FLC1* gene in *Arabidopsis* [29], *GBNSS1* gene in potato [30], and *CCA1* gene in *Arabidopsis* [12]. The chlorophyll content measured in T1 (S3 Table) and T2 (S4 Table) progeny plants showed rhythmicity with passage of time in the *CCA1* gene expression. It was at peak at 12:00 Noon when *CCA1* gene expression is lowest and again decreases until dusk (6:00 PM) and then start increasing afterwards as shown in S6 Fig. A similar correlation between *CCA1* gene expression and chlorophyll content has been reported [12] in *Arabidopsis*. However, no significant difference was observed in the level of chlorophyll content with an average of 0.04mgg^-1^ in T1 (S3 Table) and T2 (S4 Table) progeny plants having gene construct A for over expression of *CCA1* gene and RNAi constructs B and C for repression of *CCA1* gene as shown in S6 Fig.

## Conclusions

Therefore, following conclusions can be drawn from the present investigation. The over-expression of *CCA1* gene under the control of TOC1 promoter follows circadian rhythm in T1and T2 progeny plants from Constructs A of Japonica rice variety Taipei 309 has resulted in detrimental effect on morphology of progeny plants with reduced plant height, reduced number of tillers/panicles, reduced seed size and reduced thousand seed weight over the wild type. On the other hand, repression of *CCA1* gene under the control of *TOC1* promoter follows a circadian rhythm in T1and T2 transgenic progeny plants from RNAi Constructs B and C of Japonica rice variety Taipei309 has resulted in improved morphology of progeny plants with slight increase in plant height, increased number of tillers/panicles, increased yield, increased thousand seed weight and increased seed size over the wild type control which is statistically significant. Additionally, RNAi Construct–C having *CCA1* gene derived from the 3`terminal region of *CCA1* gene was found to have better morphological attributes than Construct B which was derived from 5`terminal region of *CCA1* gene. Similar results of gene silencing by RNAi vectors have been reported for *FLC1* gene in *Arabidopsis* [29], *GBNSS1* gene in potato [30], and *CCA1* gene in *Arabidopsis* [12].

Circadian clock genes including *CCA1* are potent tools to change single or multiple traits simultaneously and their potential application by exploiting clock-regulated activities is just beginning to be understood. Circadian clock genes hold the potential for crops breeding better adapted to environments and the fluctuations inherent to climate change. It is envisaged that data presented herein regarding endogenous *CCA1* gene expression studies in transgenic Japonica rice variety Taipei 309 lines and its correlation with growth vigor will significantly initiate the rice breeding programs both qualitatively and quantitatively and further help in better understanding the role of *CCA1* gene in plant growth, biomass and metabolism in future. This is the first report of successful genetic transformation of an important Japonica rice variety Taipei 309, by employing *Agrobacterium tumefaciens* strain EHA 105 harboring the plasmid vector pCAMBIA 1300 (8.959kb) having endogenous *CCA1* gene under the control of *TOC1* promoter in both over-expression and repression for studying the effect of *CCA1* gene on morphological traits in T0, T1 and T2 progeny plants.

## Supporting information

S1 Fig Modified *CCA1* cDNA sequence from rice Os08g0157600. (TIF)

S2 Fig Isolation, cloning of *TOC1* promoter, *NOS* terminator and *CCA1* gene in PUC 19. (A) *TOC1* promoter from rice genomic DNA using optimized primers showing 1.35 kb band. (B) Cloning of *TOC1* promoter in *PUC19* vector using *BamH1* & *HindIII* digest showing 1.3 kb band. (C) Amplification of *NOS* terminator gene using optimized primer showing 250 bp band. (D) Cloning of *CCA1* gene in *PUC 19* vector plasmid having *TOC1* promoter + *NOS* gene (*Sac1* and *BamHI* digest) PTCN showing 2.172 kb band.
(TIF)

S3 Fig Isolation, cloning of *CCA1a* and *CCA1b* gene fragments for construction of RNAi vectors. (A) Amplification of *CCA1a* and *CCA1b* genes using optimized primers showing 0.4kb of 5`region of *CCA1* gene and 0.395kb of 3`region of *CCA1b* gene. (B) Intermediate RNAi vector psd20 employed for cloning sense and antisense fragments of *CCA1a/b* by kicking out the *4CL* gene in sense and antisense orientation linked with a GUS linker. (C) Cloning of 0.4kb of *CCA1a* sense gene in intermediate RNAi vector psd20 by kicking out *4CL*gene in sense orientation by digesting with *BamHI*, *XhoI* and ligating *CCA1a* sense gene. (D) Cloning of 0.4kb of *CCA1a* antisense gene in above psd20 vector in which *CCA1a* sense fragment has already been moved in by kicking out *4CL*gene in antisense orientation by digesting with using *XmaI*, *SacI* restriction enzymes and ligating *CCA1a* antisense gene. (E) Cloning of 0.395kb of *CCA1b* sense gene in intermediate RNAi vector psd20 by kicking out *4CL*gene in sense orientation by digesting with *BamHI*, *XhoI* and ligating *CCA1b* sense gene. (F) Cloning of 0.395kb of *CCA1b* antisense gene in above psd20 vector in which *CCA1b* sense fragment has already been moved in by kicking out *4CL*gene in antisense orientation by digesting with using *XmaI*, *SacI* restriction enzymes and ligating *CCA1b* antisense gene.
(TIF)

S4 Fig **Morphological characteristics of T1 progeny plants at different developmental stages from germination of seeds to flowering and seed setting derived from circadian clock gene constructs A, B, & C subsequently transferred to plastic pots containing soil mixture and maintained in Transgenic Green House.** (A) The germinated transgenic plants after 7 days of sowing. (B) Transgenic progeny plants proliferating well after 2–3 weeks, producing multiple shoots. (C) Further proliferation and induction of multiple tillers in transgenic plants. (D) Flowering stage (immature panicles stage or milky stage) of transgenic progeny plants. (E) Well developed transgenic progeny plants showing further proliferation, flowering, panicle formation and seed setting and grown to maturity till harvesting.
(TIF)

S5 Fig **Morphological characteristics of T2 progeny plants at different developmental stages from germination of seeds to flowering and seed setting derived from circadian clock gene constructs A, B, & C subsequently transferred to plastic pots containing soil mixture and maintained in Transgenic Green House.** (A) The germinated transgenic plants after 7 days of sowing. (B) Transgenic progeny plants proliferating well after 2–3 weeks, producing multiple shoots. (C) Further proliferation and induction of multiple tillers and flower initiation in transgenic plants. (D) Flowering stage (immature panicles stage or milky stage) of transgenic progeny plants. (E) Well developed transgenic progeny plants showing further proliferation, flowering, panicle formation and seed setting and grown to maturity till harvesting.
(TIF)

S6 Fig Quantification of chlorophyll content in T1 and T2 transgenic progeny plants at different time points; 6:00AM, 12:00 Noon, 6:00 PM and 9:00 AM (the following day). (A) T1 transgenic progeny plants. (B) T2 transgenic progeny plants.
(TIF)

S7 Fig Full length images of gel pictures of PCR analysis of T1 transgenic plants for the presence of the *hyg* gene as shown in Fig 5B. (JPG)

S1 Table Comparison of seed size of T1 transgenic progeny plants harboring gene constructs *A*, *B* and *C* and that of wild type (WT).
(DOC)

S2 Table Comparison of seed size of T2 transgenic progeny plants harboring gene constructs *A*, *B* and *C* and that of wild type (WT).
(DOC)

S3 Table Comparison of average chlorophyll content (mgg-1) in T1 transgenic progeny plants at different time points; 6:00AM, 12:00 Noon, 6:00 PM and 9:00 AM the following day. (DOC)

S4 Table Comparison of average chlorophyll content (mgg-1) in T2 transgenic progeny plants at different time points; 6:00AM, 12:00 Noon, 6:00 PM and 9:00 AM the following day. (DOC)

## Abbreviations

*CCA1*gene: Circadian Clock Associated 1 gene
*hyg* gene: Hygromycin resistance gene
2,4-D: 2,4,Dichlorophenoxy acetic acid
BAP: 6 Benzyl amino purine
