# Supplementary figures and images for: Isolation, cloning and expression of CCA1 gene in transgenic progeny plants of Japonica rice exhibiting altered morphological traits
Source: PLoS One. 2019 Aug 5;14(8):e0220140. doi: 10.1371/journal.pone.0220140 (PMC6681968; doi:10.1371/journal.pone.0220140)

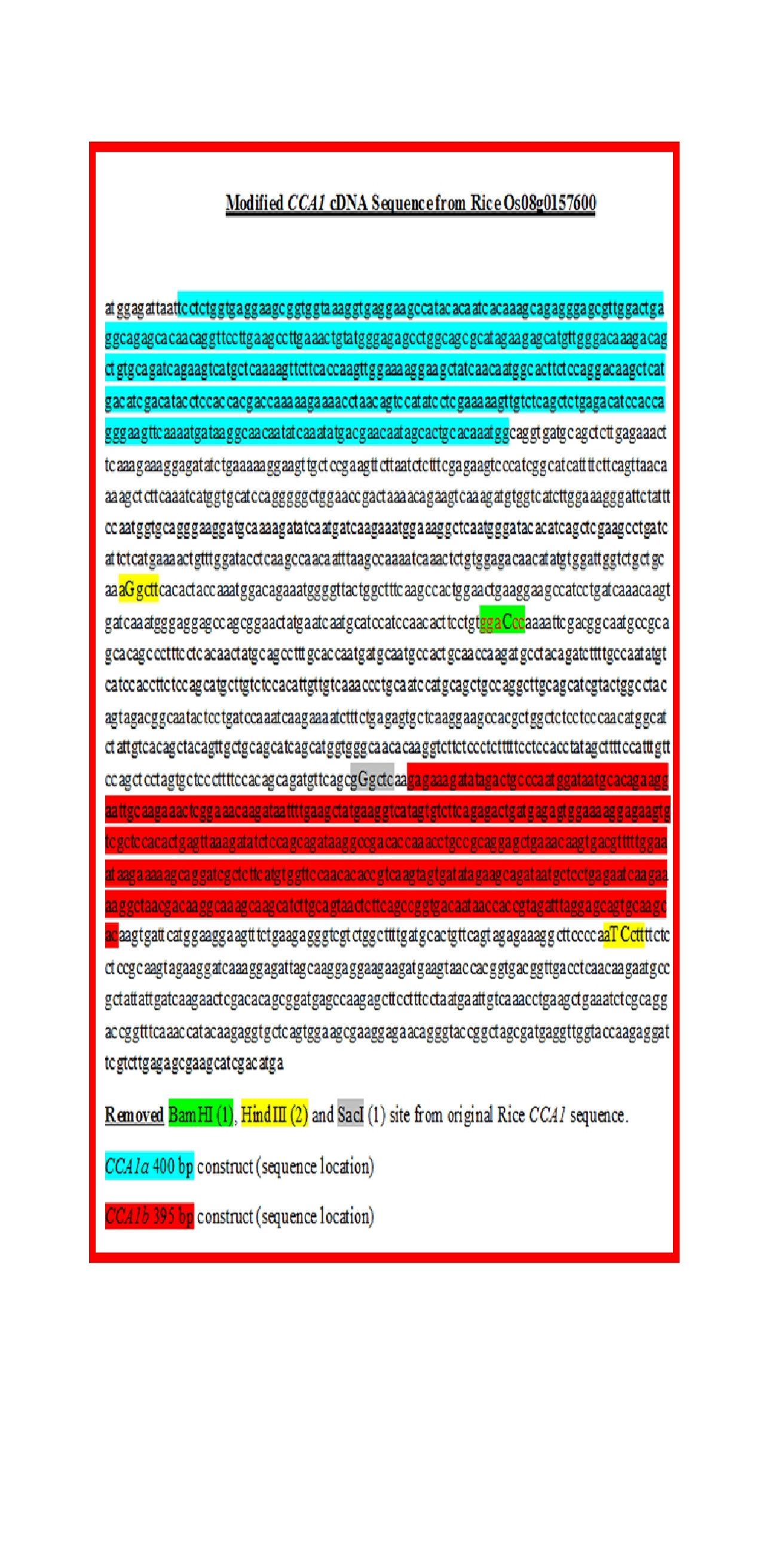

Supplement: S1 Fig — (TIF) [file pone.0220140.s001.tif]

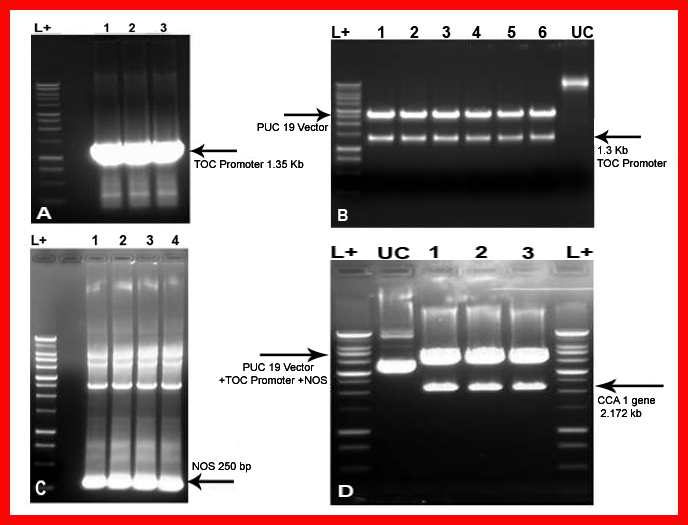

Supplement: S2 Fig — (A) TOC1 promoter from rice genomic DNA using optimized primers showing 1.35 kb band. (B) Cloning of TOC1 promoter in PUC19 vector using BamH1 & HindIII digest showing 1.3 kb band. (C) Amplification of NOS terminator gene using optimized primer showing 250 bp band. (D) Cloning of CCA1 gene in PUC 19 vector plasmid having TOC1 promoter + NOS gene (Sac1 and BamHI digest) PTCN showing 2.172 kb band. (TIF) [file pone.0220140.s002.tif]

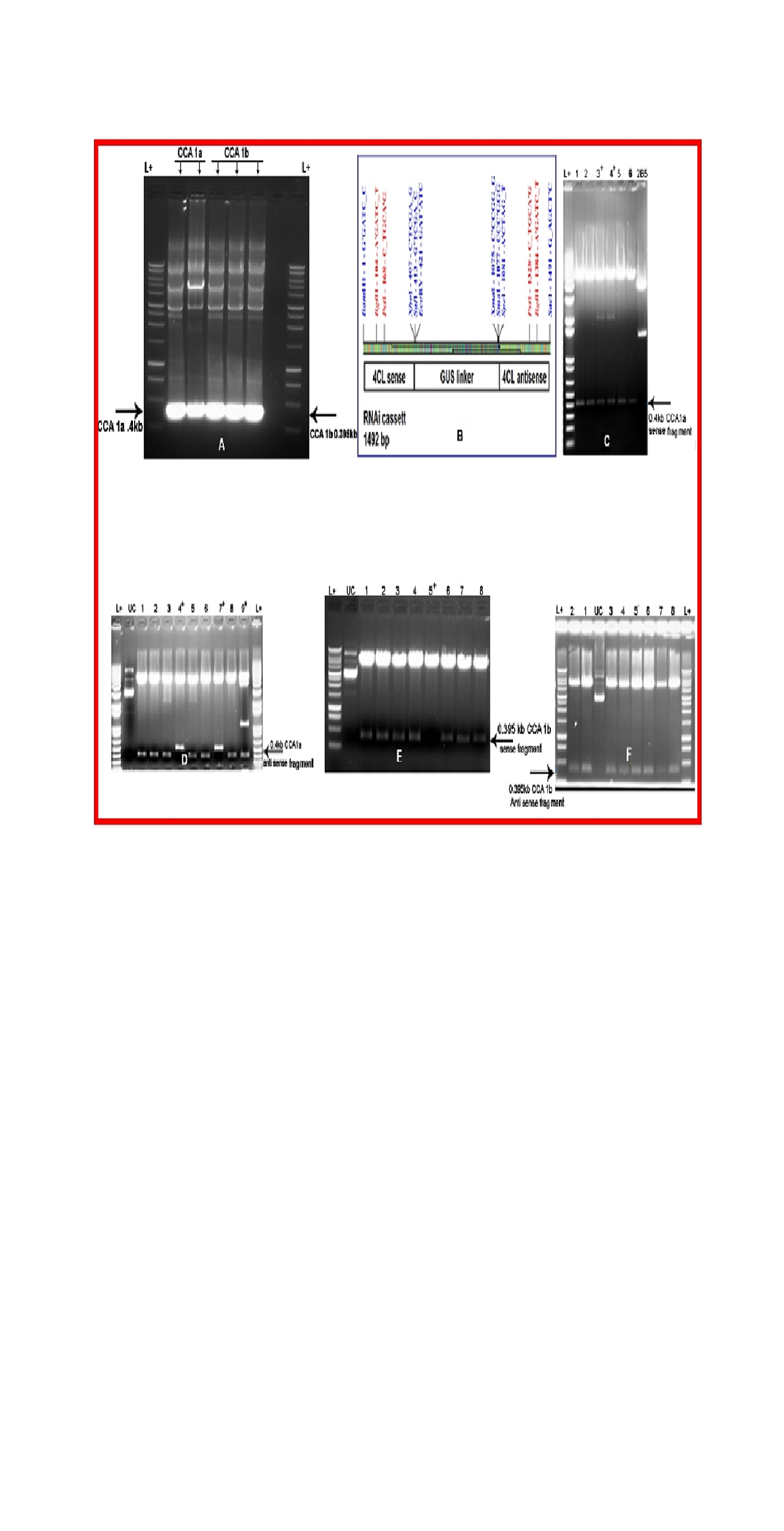

Supplement: S3 Fig — (A) Amplification of CCA1a and CCA1b genes using optimized primers showing 0.4kb of 5`region of CCA1 gene and 0.395kb of 3`region of CCA1b gene. (B) Intermediate RNAi vector psd20 employed for cloning sense and antisense fragments of CCA1a/b by kicking out the 4CL gene in sense and antisense orientation linked with a GUS linker. (C) Cloning of 0.4kb of CCA1a sense gene in intermediate RNAi vector psd20 by kicking out 4CLgene in sense orientation by digesting with BamHI, XhoI and ligating CCA1a sense gene. (D) Cloning of 0.4kb of CCA1a antisense gene in above psd20 vector in which CCA1a sense fragment has already been moved in by kicking out 4CLgene in antisense orientation by digesting with using XmaI, SacI restriction enzymes and ligating CCA1a antisense gene. (E) Cloning of 0.395kb of CCA1b sense gene in intermediate RNAi vector psd20 by kicking out 4CLgene in sense orientation by digesting with BamHI, XhoI and ligating CCA1b sense gene. (F) Cloning of 0.395kb of CCA1b antisense gene in above psd20 vector in which CCA1b sense fragment has already been moved in by kicking out 4CLgene in antisense orientation by digesting with using XmaI, SacI restriction enzymes and ligating CCA1b antisense gene. (TIF) [file pone.0220140.s003.tif]

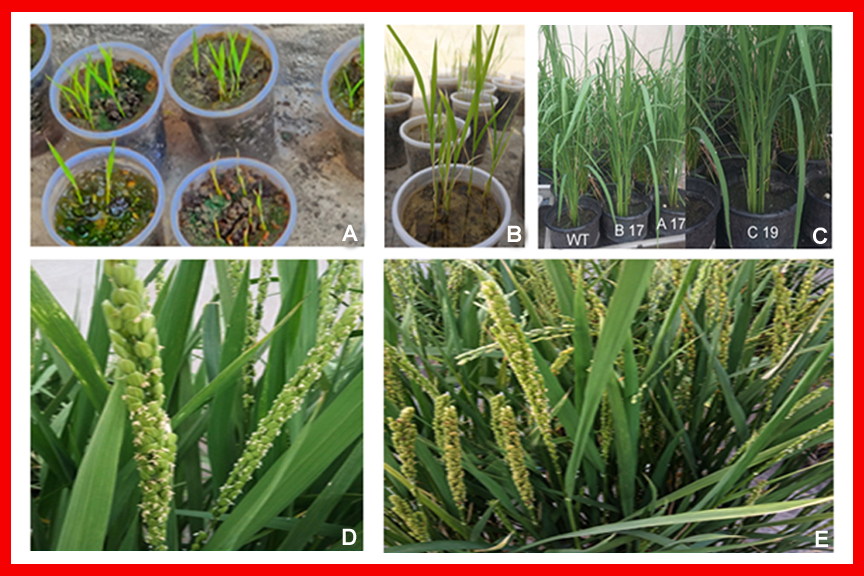

Supplement: S4 Fig — Morphological characteristics of T1 progeny plants at different developmental stages from germination of seeds to flowering and seed setting derived from circadian clock gene constructs A, B, & C subsequently transferred to plastic pots containing soil mixture and maintained in Transgenic Green House. (A) The germinated transgenic plants after 7 days of sowing. (B) Transgenic progeny plants proliferating well after 2–3 weeks, producing multiple shoots. (C) Further proliferation and induction of multiple tillers in transgenic plants. (D) Flowering stage (immature panicles stage or milky stage) of transgenic progeny plants. (E) Well developed transgenic progeny plants showing further proliferation, flowering, panicle formation and seed setting and grown to maturity till harvesting. (TIF) [file pone.0220140.s004.tif]

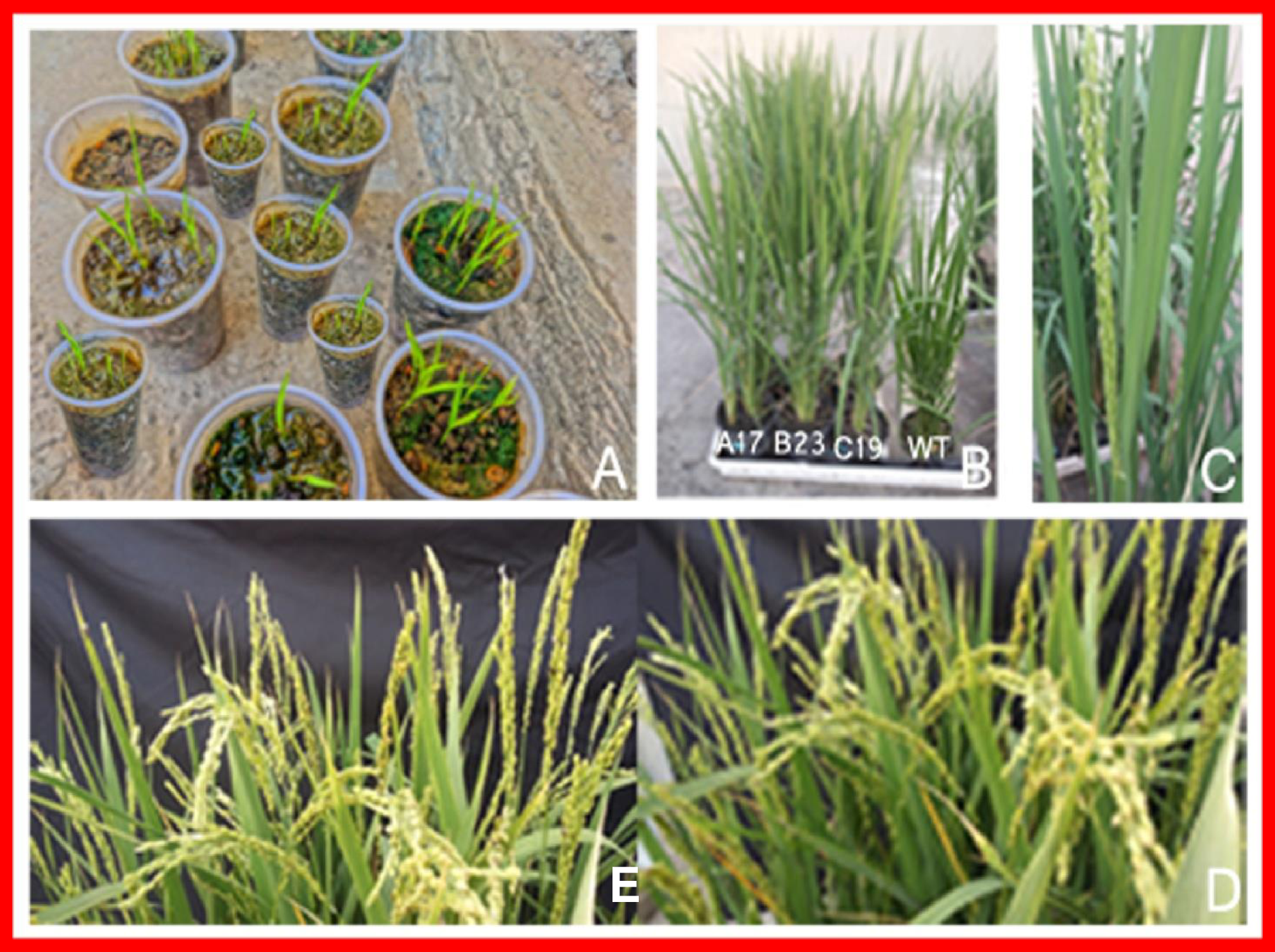

Supplement: S5 Fig — Morphological characteristics of T2 progeny plants at different developmental stages from germination of seeds to flowering and seed setting derived from circadian clock gene constructs A, B, & C subsequently transferred to plastic pots containing soil mixture and maintained in Transgenic Green House. (A) The germinated transgenic plants after 7 days of sowing. (B) Transgenic progeny plants proliferating well after 2–3 weeks, producing multiple shoots. (C) Further proliferation and induction of multiple tillers and flower initiation in transgenic plants. (D) Flowering stage (immature panicles stage or milky stage) of transgenic progeny plants. (E) Well developed transgenic progeny plants showing further proliferation, flowering, panicle formation and seed setting and grown to maturity till harvesting. (TIF) [file pone.0220140.s005.tif]

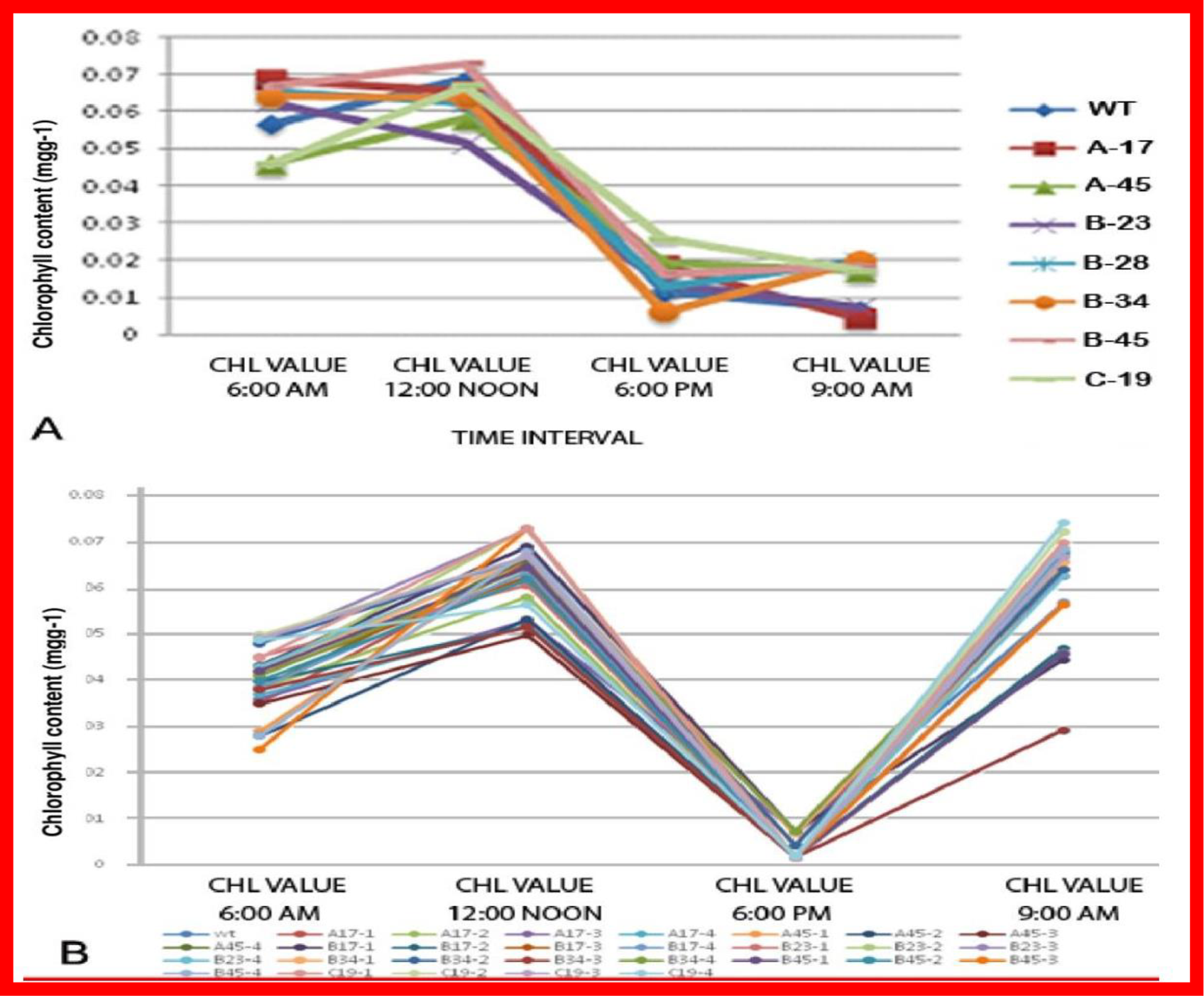

Supplement: S6 Fig — (A) T1 transgenic progeny plants. (B) T2 transgenic progeny plants. (TIF) [file pone.0220140.s006.tif]

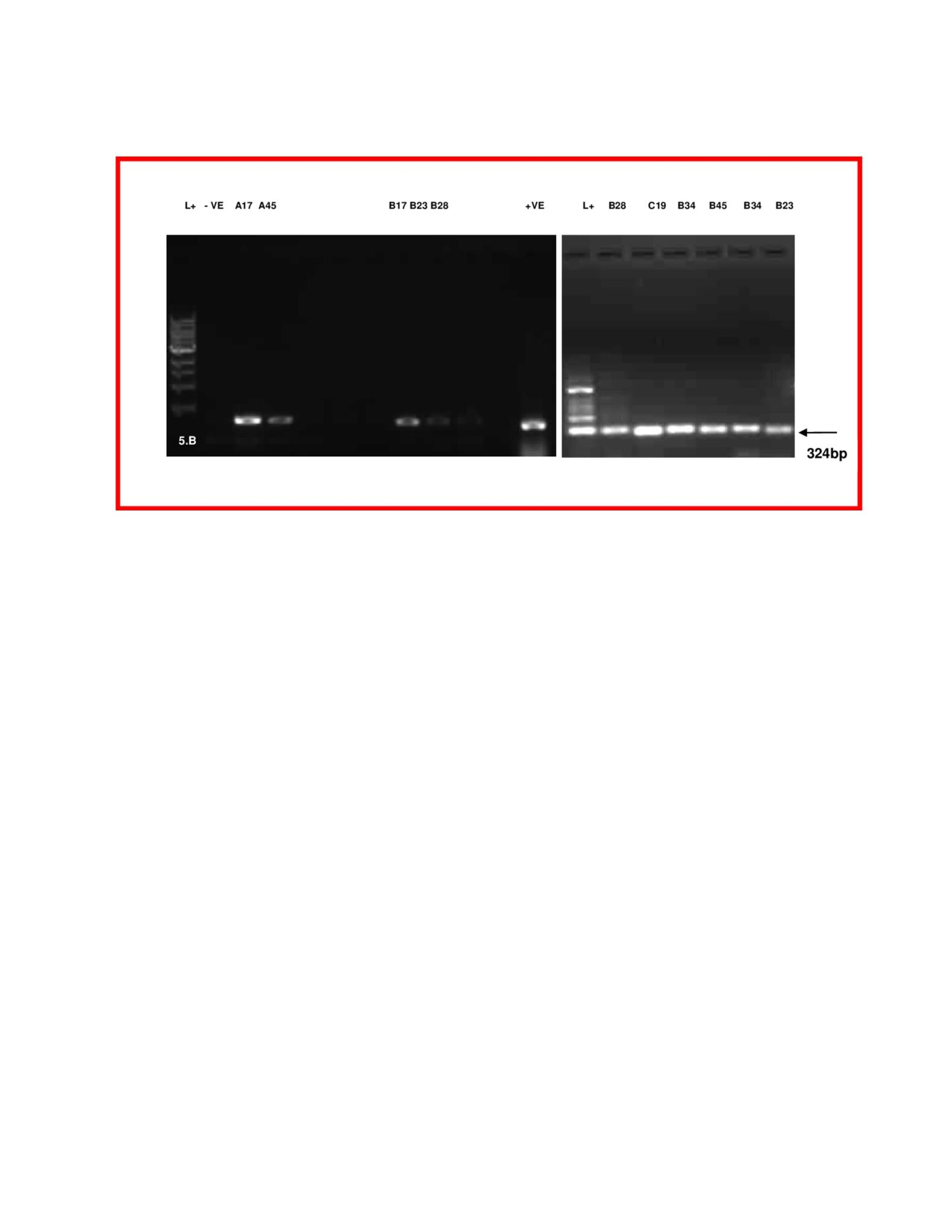

Supplement: S7 Fig — (JPG) [file pone.0220140.s007.jpg]
