# Supplementary material for: Isolation, cloning and expression of CCA1 gene in transgenic progeny plants of Japonica rice exhibiting altered morphological traits
Source: PLoS One. 2019 Aug 5;14(8):e0220140. doi: 10.1371/journal.pone.0220140 (PMC6681968; doi:10.1371/journal.pone.0220140)
Supplement: S1 Table — Comparison of seed size of T1 transgenic progeny plants harboring gene constructs A, B and C and that of wild type (WT). (DOC) [file pone.0220140.s008.doc]

**S1 Table. Comparison of seed size of T1 transgenic progeny plants harboring gene constructs *A*, *B* and *C* and that of wild type (WT).**

| **T1 Transgenic Progeny Lines** | **Average Seed Length (mm)** | **Average Seed Width (mm)** |
| --- | --- | --- |
| WT | 6.75 | 1.80 |
| A-17 | 5.5 | 1.30 |
| A-45 | 5.4 | 1.75 |
| B-17 | 7.02 | 2.01 |
| B-23 | 6.98 | 1.98 |
| B-28 | 7.02 | 2.0 |
| B-34 | 6.98 | 2.03 |
| B-45 | 7.01 | 2.05 |
| C-19 | 7.43 | 2.18 |
