# Supplementary material for: Isolation, cloning and expression of CCA1 gene in transgenic progeny plants of Japonica rice exhibiting altered morphological traits
Source: PLoS One. 2019 Aug 5;14(8):e0220140. doi: 10.1371/journal.pone.0220140 (PMC6681968; doi:10.1371/journal.pone.0220140)
Supplement: S2 Table — Comparison of seed size of T2 transgenic progeny plants harboring gene constructs A, B and C and that of wild type (WT). (DOC) [file pone.0220140.s009.doc]

**S2Table. Comparison of seed size of T2 transgenic progeny plants harboring gene constructs *A*, *B* and *C* and that of wild type (WT).**

| **T1 Transgenic Progeny Lines** | **Average Seed Length (mm)** | **Average Seed Width (mm)** |
| --- | --- | --- |
| WT | 6 | 1.7 |
| A17-1 | 5 | 1.1 |
| A17-2 | 5.3 | 1.3 |
| A17-3 | 5.8 | 1.5 |
| A17-4 | 5.3 | 1.7 |
| A45-1 | 4.98 | 1.6 |
| A45-2 | 5.2 | 1.3 |
| A45-3 | 6.5 | 1.5 |
| A45-4 | 6.58 | 1.8 |
| B17-1 | 7.2 | 1.7 |
| B17-2 | 6.8 | 2.3 |
| B17-3 | 6.9 | 2.03 |
| B17-4 | 5.8 | 1.9 |
| B23-1 | 6.5 | 1.7 |
| B23-2 | 7.2 | 1.8 |
| B23-2 | 6.1 | 2.5 |
| B23-3 | 6.98 | 2.0 |
| B34-4 | 7.1 | 2.0 |
| B34-1 | 6.5 | 1.8 |
| B34-2 | 6.3 | 1.7 |
| B34-3 | 5.8 | 1.9 |
| B45-4 | 7 | 1.6 |
| B45-1 | 6.6 | 1.8 |
| B45-2 | 7.1 | 1.7 |
| B45-3 | 7.43 | 2.4 |
| C19-1 | 7.5 | 2.98 |
| C19-2 | 6.9 | 2.98 |
| C19-3 | 7.5 | 2.5 |
| C19-4 | 5.7 | 2.9 |
