# Supplementary material for: Isolation, cloning and expression of CCA1 gene in transgenic progeny plants of Japonica rice exhibiting altered morphological traits
Source: PLoS One. 2019 Aug 5;14(8):e0220140. doi: 10.1371/journal.pone.0220140 (PMC6681968; doi:10.1371/journal.pone.0220140)
Supplement: S3 Table — (DOC) [file pone.0220140.s010.doc]

**S3 Table. Comparison of average chlorophyll content (mgg^-1^) in T1 transgenic progeny plants at different time points; 6:00AM, 12:00 Noon, 6:00 PM and 9:00 AM the following day.**

| **T1 Transgenic Progeny Lines** | **6 AM** | **12 Noon** | **6 PM** | **9 AM** |
| --- | --- | --- | --- | --- |
| WT | 0.0385 | 0.0392 | 0.042 | 0.0444 |
| A17 | 0.0344 | 0.0351 | 0.0361 | 0.0445 |
| A45 | 0.0371 | 0.0381 | 0.0416 | 0.0444 |
| B17 | 0.0406 | 0.432 | 0.0417 | 0.0418 |
| B23 | 0.0423 | 0.0398 | 0.043 | 0.0415 |
| B28 | 0.0383 | 0.0415 | 0.0379 | 0.0423 |
| B34 | 0.0333 | 0.0385 | 0.0409 | 0.0451 |
| B45 | 0.0361 | 0.0452 | 0.0323 | 0.0447 |
| C19 | 0.0354 | 0.0492 | 0.0315 | 0.0448 |
