# Supplementary material for: Isolation, cloning and expression of CCA1 gene in transgenic progeny plants of Japonica rice exhibiting altered morphological traits
Source: PLoS One. 2019 Aug 5;14(8):e0220140. doi: 10.1371/journal.pone.0220140 (PMC6681968; doi:10.1371/journal.pone.0220140)
Supplement: S4 Table — (DOC) [file pone.0220140.s011.doc]

**S4 Table. Comparison of average chlorophyll content (mgg^-1^) in T2 transgenic progeny plants at different time points; 6:00AM, 12:00 Noon, 6:00 PM and 9:00 AM the following day.**

| **T2 Transgenic Progeny Lines** | **6 AM** | **12 Noon** | **6 PM** | **9 AM** |
| --- | --- | --- | --- | --- |
| WT | 0.043 | 0.0689 | 0.00674 | 0.0564 |
| A-17-1 | 0.0351 | 0.0655 | 0.00427 | 0.0685 |
| A-17-2 | 0.0381 | 0.058 | 0.0017 | 0.0459 |
| A-17-3 | 0.036 | 0.053 | 0.00736 | 0.06259 |
| A-17-4 | 0.037 | 0.0521 | 0.00197 | 0.0656 |
| A-45-1 | 0.029 | 0.0667 | 0.00197 | 0.064 |
| A-45-2 | 0.028 | 0.0533 | 0.00172 | 0.0292 |
| A-45-3 | 0.035 | 0.0498 | 0.00183 | 0.0669 |
| A-45-4 | 0.0383 | 0.0663 | 0.00169 | 0.0458 |
| B-17-1 | 0.0432 | 0.0689 | 0.00736 | 0.0444 |
| B-17-2 | 0.0398 | 0.0516 | 0.00197 | 0.0469 |
| B-17-3 | 0.0415 | 0.0625 | 0.00199 | 0.0567 |
| B-17-4 | 0.0385 | 0.0636 | 0.00174 | 0.0568 |
| B-23-1 | 0.0452 | 0.0607 | 0.00166 | 0.0564 |
| B-23-2 | 0.04164 | 0.0729 | 0.00169 | 0.0685 |
| B-23-3 | 0.048 | 0.0728 | 0.00173 | 0.0459 |
| B-23-4 | 0.043 | 0.0669 | 0.00169 | 0.06259 |
| B-28-1 | 0.0445 | 0.0665 | 0.00169 | 0.0635 |
| B-28-2 | 0.0382 | 0.0668 | 0.00185 | 0.0655 |
| B-28-3 | 0.0419 | 0.0728 | 0.00182 | 0.0559 |
| B-28-4 | 0.045 | 0.0648 | 0.0174 | 0.0453 |
| B-34-1 | 0.042 | 0.0668 | 0.00674 | 0.0656 |
| B-34-2 | 0.048 | 0.0668 | 0.00427 | 0.064 |
| B-34-3 | 0.038 | 0.0516 | 0.00173 | 0.0292 |
| B-34-4 | 0.041 | 0.0645 | 0.00736 | 0.0669 |
| B-45-1 | 0.0421 | 0.0646 | 0.00197 | 0.0458 |
| B-45-2 | 0.0398 | 0.0618 | 0.00199 | 0.0678 |
| B-45-3 | 0.025 | 0.0729 | 0.00174 | 0.0564 |
| B-45-4 | 0.028 | 0.0679 | 0.00183 | 0.0685 |
| C-19-1 | 0.045 | 0.0729 | 0.00169 | 0.0698 |
| C-19-2 | 0.0498 | 0.0669 | 0.00199 | 0.0721 |
| C-19-3 | 0.0492 | 0.0669 | 0.00171 | 0.0666 |
| C-19-4 | 0.0487 | 0.0564 | 0.00193 | 0.0741 |
